# Global asymmetries in the moisture origins of atmospheric river precipitation

**DOI:** 10.1038/s41612-026-01408-6

**Published:** 2026-04-08

**Authors:** Alfredo Crespo-Otero, Damián Insua-Costa, Victoria M. H. Deman, Emilio Hernández-García, Cristóbal López, Gonzalo Míguez-Macho

**Affiliations:** 1 CRETUS, Non-linear Physics Group, Universidade de Santiago de Compostela, Santiago de Compostela, Spain; 2 Hydro-Climate Extremes Lab (H-Cel), Ghent University, Ghent, 9000 Belgium; 3 Instituto de Física Interdisciplinar y Sistemas Complejos (IFISC), CSIC-UIB, Campus Universitat de les Illes Balears, 07122 Palma de Mallorca, Spain

**Keywords:** Climate sciences, Hydrology

## Abstract

Atmospheric rivers (ARs) are key agents of poleward heat and moisture transport, yet the extent to which tropical moisture directly feeds AR precipitation at higher latitudes remains debated. Here we present a forty-year global climatology of moisture sources for AR-related precipitation, combining deep learning-based AR detection with Lagrangian moisture tracking. We reveal that AR precipitation worldwide organizes into two coherent and contrasting moisture transport regimes. Along western continental boundaries, AR precipitation is predominantly sustained by extratropical moisture convergence, with tropical contributions typically limited to 30–40%, whereas eastern boundaries exhibit shorter transport pathways with substantially larger tropical contributions, averaging 60–70%. These persistent west–east asymmetries reflect systematic shifts in moisture origin as ARs evolve from early subtropical acquisition to mature extratropical transport. We further show a strong negative correlation between tropical contribution and latitude, demonstrating that direct tropical moisture delivery is not a requirement for AR precipitation at high latitudes. Instead, tropical moisture primarily preconditions AR formation, while the water mass that ultimately precipitates is progressively replaced along the poleward pathway. Taken together, our findings reconcile divergent assessments of tropical moisture influence on AR precipitation and thus deepen our understanding of how Earth’s hydrological engine redistributes atmospheric water across the planet.

## Introduction

Atmospheric rivers (ARs) are long, narrow, and transient corridors of enhanced water vapor transport that occur frequently over mid-latitude ocean basins^1,2^. They account for more than 90% of the poleward moisture transport in these regions^3,4^ and typically extend for several thousand kilometers while remaining embedded in the lower troposphere, often within the warm sector of extratropical cyclones. Their influence spans a broad range of hydroclimatic environments: in mid-latitudes, AR landfalls are a leading source of heavy precipitation, shaping seasonal water availability and hydrological extremes in regions such as the western United States and western Europe^5–9^. Closely related to their role in extreme rainfall, ARs have been shown to drive flooding in the world’s major river basins and exert a substantial influence on surface water resources and groundwater recharge, although their hydrological impacts can vary depending on basin characteristics and antecedent conditions^10–14^. Recent work has also associated AR activity with warm winters and extreme heat events^15^, and at high latitudes, AR intrusions have been linked to strong warm anomalies and major melting events, both in northeast Greenland^16^ and in West Antarctica^17^.

A key open question concerns the origin of the water vapor that ultimately produces precipitation within ARs. Their frequent direct physical connection with the moist atmosphere in the tropical belt, as they seemingly stem from its winding margin^18^, suggests substantial long-range transport, yet the relative importance of tropical and extratropical sources remains debated. Some studies identify moisture uptake primarily in mid-latitude oceans, emphasizing extratropical evaporation and local convergence^19–21^, whereas others highlight a stronger tropical influence^22–24^. The role of terrestrial moisture recycling is also unclear. Continental evapotranspiration has been shown to fuel ARs in specific regions, including the Arctic and southeastern Asia^25,26^, while interactions with low-level jets such as the South American Low Level Jet (SALLJ) can influence AR maintenance and moisture transport over South America and the South Atlantic^27^. Consequently, uncertainty persists regarding the relative contributions of tropical and extratropical, as well as oceanic and terrestrial, source regions sustaining AR-related precipitation.

Resolving this uncertainty is essential for understanding how AR-related precipitation responds to variability in the ocean–atmosphere system, including seasonality and departures from long-term mean conditions, and for constraining the mechanisms that shape AR behavior across climates. The location and characteristics of moisture source regions influence the thermodynamic evolution of ARs, their sensitivity to modes of variability such as the El Niño-Southern Oscillation (ENSO) or the Madden-Julian Oscillation (MJO), which can alter ARs’ seasonal distribution and intensity, and the coherence and predictability of their upstream pathways. They also modulate the amount of heat and moisture transported into mid and high latitudes, conditioning both precipitation and heat extremes. The scarcity of observational data, especially vertically resolved moisture measurements over oceans and remote continental regions, has led most AR and moisture tracking studies to rely too heavily on reanalysis and modeling frameworks. Progress has been limited by the regional focus of most studies, mainly western North America and western Europe, and by methodological differences among Eulerian and Lagrangian tracking approaches, which often yield divergent results^28–30^. Nevertheless, key moisture source areas sustaining AR-related precipitation have been identified for some regions^20,21,31,32^, although the relative importance of the different sources is not generally quantified, and the global organization of these sources is not investigated, leaving the debate unresolved.

Here, we address these limitations by constructing a global climatology of moisture sources for precipitation in ARs. We combine a deep learning AR detection algorithm capable of identifying ARs worldwide^33,34^ with a Lagrangian moisture tracking framework based on FLEXPART v10.4^35^ and the Dirmeyer and Brubaker (1999) methodology^36^, previously validated against the WRF with Water Vapor Tracers model^29,37^. Unlike previous regional or case-based studies, our analysis provides a globally consistent, event-based quantification of moisture contributions to precipitation across all major AR-affected regions. By analyzing air parcel trajectories over 1980–2023, we identify coherent large-scale patterns in moisture origin that reflect the global regime of water vapor transport associated with ARs.

## Results

### Global patterns of atmospheric river footprints

**Fig. 1:**
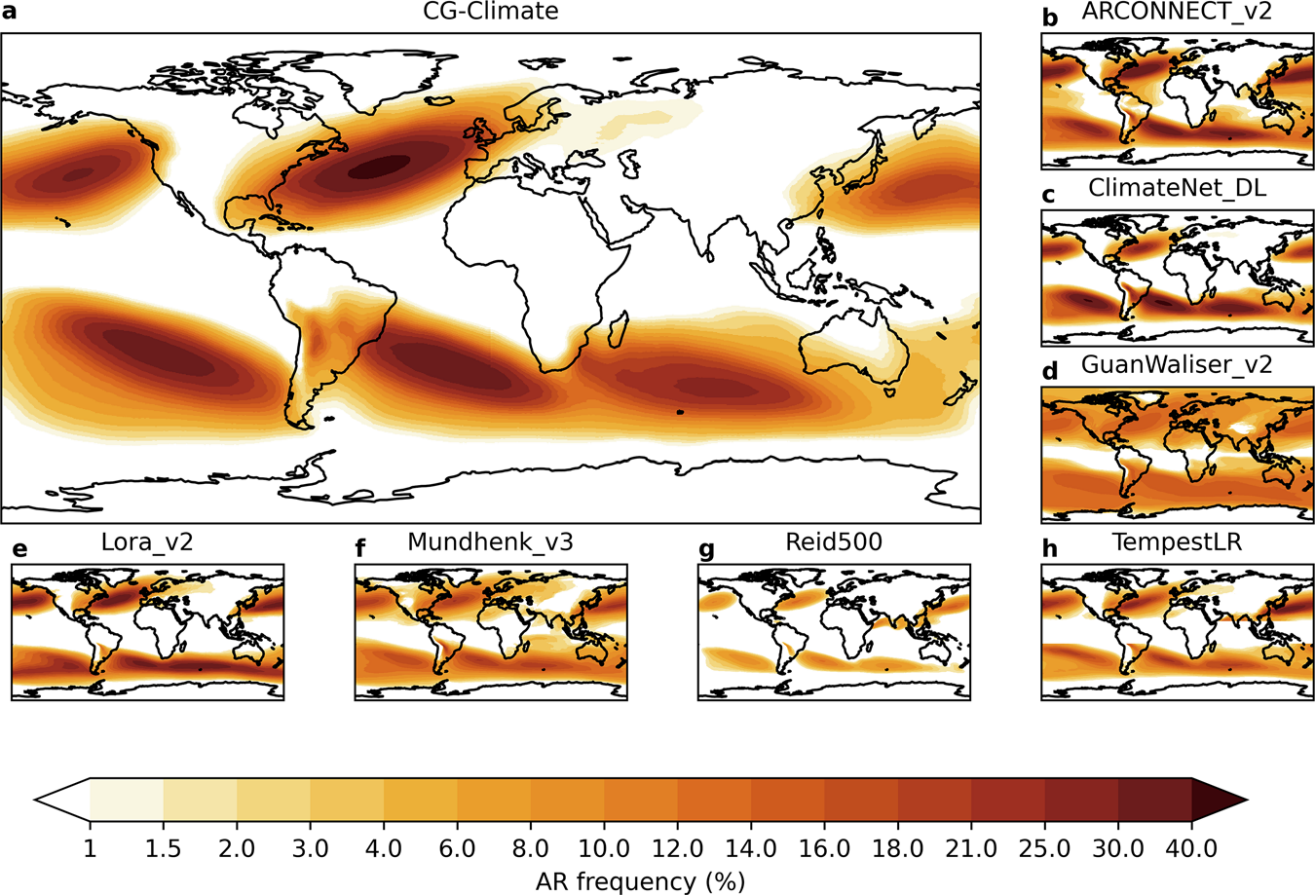
AR detection frequency. AR frequency for the DL algorithm used in this study (CG-Climate, **a**) and for the rest of algorithms participating in the ARTMIP project Tier 2 with global coverage (**b**−**h**), during the period 2000−2019.

**Fig. 2:**
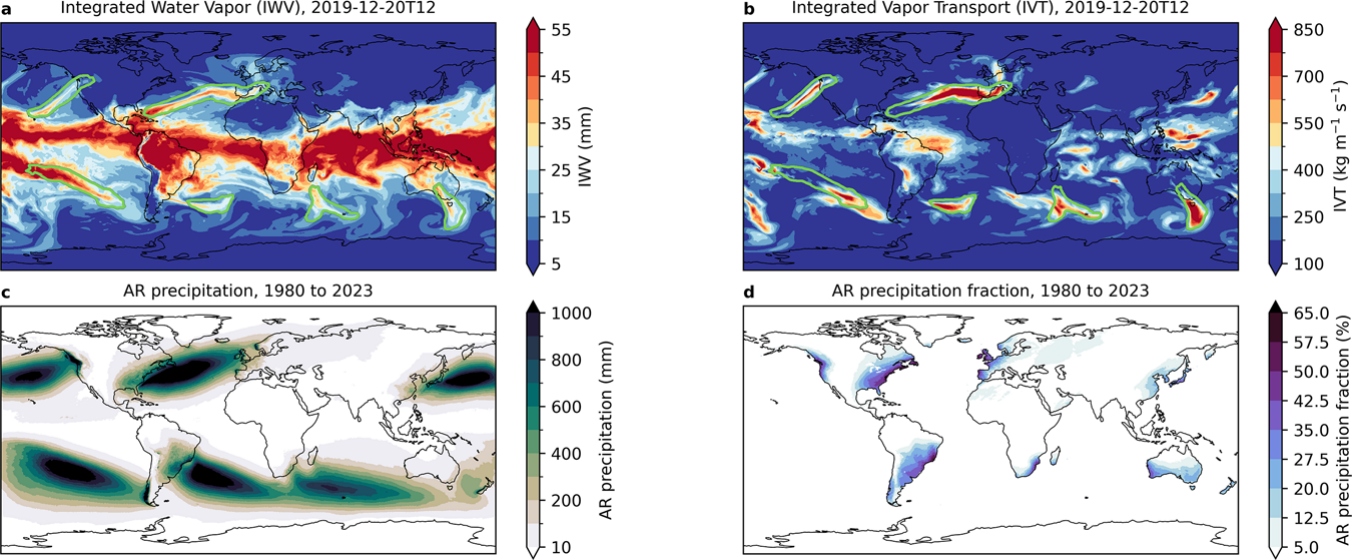
Example of detection and AR-related precipitation. AR detections (green contours) together with integrated water vapor (**a**) and integrated vapor transport (**b**) on 2019-12-20T12. Average annual precipitation attributable to ARs from 1980 to 2023 (**c**) and its percentage of total precipitation over land (**d**).

Figure 1 shows the AR frequency (percentage of time steps with AR detections) obtained with CG-Climate, the detection algorithm used in this study (Fig. 1a), and with all algorithms participating in the Atmospheric River Tracking Method Intercomparison Project (ARTMIP Tier 2)^38^ that provide global coverage (Fig. 1b-h). To ensure consistency, results are restricted to their common period (2000–2019) and all detections are based on ERA5^39^. Except for GuanWaliser_v2 (Fig. 1d) and, to a lesser extent, Mundhenk_v3 (Fig. 1f), all algorithms show substantially higher AR frequency over the extratropical ocean basins, consistent with the climatological storm-track activity maxima. This close correlation highlights the correspondence between ARs and the warm conveyor belts of some extratropical cyclones^40^. Nevertheless, despite the broad consensus among AR detection algorithms, Fig. 1 reveals also significant variations in their results. The AR pattern from CG-Climate most closely resembles that of ClimateNet_DL—expected because both are deep-learning models trained on the same dataset but with different architectures^33,41^—and is also similar to ARCONNECT_v2 and Lora_v2 in both spatial distribution and magnitude. The differences with GuanWaliser and Reid500 are notable, as these are more restrictive AR detection algorithms, imposing high thresholds on the IVT climatology and absolute values, respectively. Figure 1 clearly shows that CG-Climate produces the largest maxima in AR frequency, reflecting its tendency to detect ARs with larger spatial extent^34^, which becomes apparent when examining specific case detections (Fig. 2a–b). These larger AR footprints do not reflect false positives but rather a potential positive bias in the area of the structures identified by the algorithm. Thus, many of the additional precipitation events identified by CG-Climate are physically consistent with AR-related moisture transport, even if they fall outside the AR mask defined by other detection methodologies.

The global distribution of average annual AR-related precipitation for 1980−2023 (Fig. 2c) closely mirrors the spatial pattern of AR frequency, with the largest totals occurring over the mid-latitude oceans (Supplementary Fig. 1 shows the corresponding fields obtained using the ARTMIP detection algorithms). Over land, however, strong regional contrasts emerge. On the Pacific coasts of North America and Chile, annual AR-related precipitation exceeds 1000 mm due to orographic lift in the existing alongshore cordilleras, but decreases sharply inland in their rain shadow. In western Europe, AR-related precipitation surpasses 500 mm in regions such as western Norway, Ireland, and northwestern Iberia, and inland impacts extend farther than in America due to the absence of major coastal mountain ranges. Beyond these well-known hotspots, Fig. 2c also highlights other regions with substantial contributions from ARs: eastern North and South America, eastern South Africa, eastern China, Japan, and New Zealand all receive more than 300 mm of AR-related precipitation across extensive areas. When expressed as a fraction of total precipitation (Fig. 2d), AR contributions generally range from 20% to 40% across these regions, with peak values surpassing 50%, although with considerable spatial variability (see Supplementary Fig. 2 for ARTMIP-based results). In areas with very high annual rainfall, such as southern Chile, large amounts of AR precipitation can represent modest relative contributions ( ~ 20%), whereas in arid regions such as western Australia the fraction exceeds 35%, meaning that even 100−200 mm of AR-related rainfall can represent a hydrologically significant share of the local water budget.

### Moisture source climatology in atmospheric river hotspot regions: western North America and western Europe

**Fig. 3:**
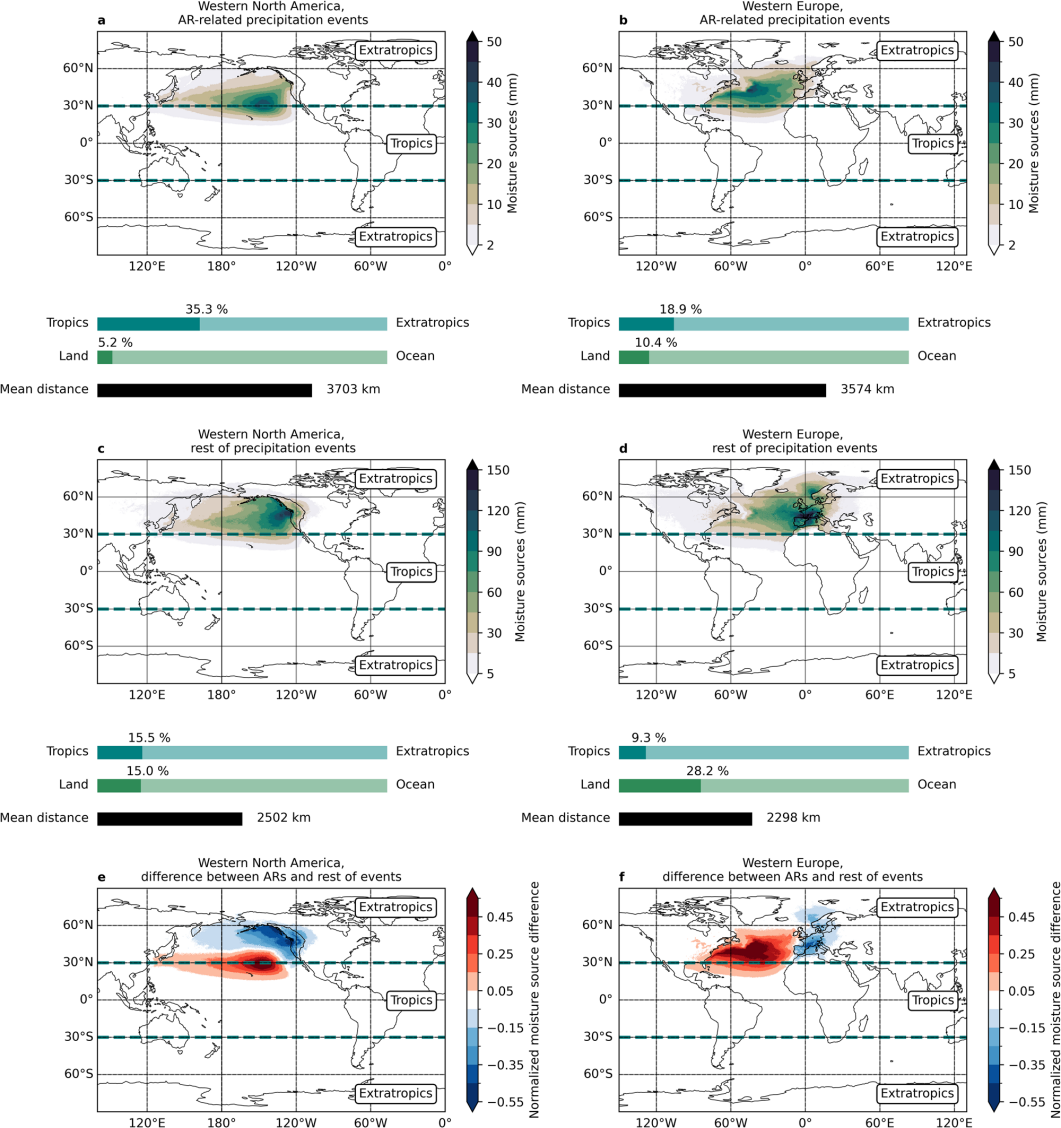
Precipitation sources in AR hotspot regions. Mean annual accumulated moisture source fields (color maps) and associated metrics (bars): mean tropical and continental contributions, and mean source-sink distance, for precipitation events affecting western North America (**a**, **c**) and western Europe (**b**, **d**), from 1980 to 2023. Panels **a** and **c** correspond to AR-related precipitation, whereas **b** and **d** represent non-AR events. The normalized difference between the moisture source fields is shown in panel **e** for western North America and panel **f** for western Europe.

Moisture sources were computed for all precipitation events associated with ARs exceeding 10 mm per day. For comparison, we also derived moisture sources for a large sample of non-AR precipitation events in all regions where AR-related rainfall is non-negligible (see Methods). Figure 3 shows the mean annual accumulated moisture sources for AR-related precipitation (Fig. 3a,b) and non-AR precipitation (Fig. 3c,d) in two canonical AR hotspot regions: western North America (9089 AR and 5832 non-AR daily events) and western Europe (12582 AR and 5854 non-AR events). Black contours delineate the target regions used in the analysis. To facilitate interpretation, we further quantify three metrics: the tropical contribution (fraction of precipitation originating from 30° S-30° N), the continental contribution, and the mean source–sink distance across events (see Methods). Distinct and consistent contrasts emerge between AR and non-AR precipitation in both hotspots: ARs draw moisture from substantially more remote regions, reflected in source–sink distances exceeding those of non-AR events by over 1000 km, and exhibit larger tropical and smaller continental contributions. Yet extratropical regions remain the dominant suppliers, indicating that ARs, despite most often emerging from humid tropical air masses, are replenished continuously along their trajectories rather than deriving their moisture from a single distant reservoir.

In western North America, moisture primarily originates from across the North Pacific during both AR and non-AR precipitation events (Fig. 3a,c,e). For non-AR events, the moisture source field extends markedly inland, yielding a comparatively high continental contribution of 15.0%, compared with only 5.2% for AR-related precipitation. During AR events, however, the maximum in the moisture source field shifts westward and, more prominently, southward—below the typical position of the Pineapple Express, a major pathway of North Pacific ARs affecting the west coast of North America^42^. This southward displacement results in a pronounced tropical contribution (35.3%) and a substantially greater mean source–sink distance ( ~ 3700 km) compared with non-AR events. These tropical fractions are consistent with previous work reporting contributions ranging from 25% to 50% for the most intense ARs, based on analyses of 29 extreme AR events affecting western North America^20^.

In western Europe (Fig. 3b,d,f), the moisture source field for AR-related precipitation extends further eastward and slightly northward relative to western North America, reflecting the weaker meridional component of ARs making landfall in Europe (see Fig. 2a,b). A distinguishing feature of this hotspot is the larger continental contribution (10.4% vs 5.2% in western North America), which may originate from both local sources, such as evapotranspiration over southwestern Europe transported downwind by ARs impacting central Europe, and remote continental sources, primarily from evaporation over eastern North America (Fig. 3c). Despite these regional differences, the core contrast between AR and non-AR precipitation remains robust: ARs draw moisture from more distant regions and exhibit larger tropical (18.9% vs 9.3%) and smaller continental (10.4% vs 28.2%) contributions. These patterns are consistent with previous work showing that ARs affecting western Europe uptake moisture over a broad Atlantic corridor extending from the Florida Peninsula along the North Atlantic storm track^21^. While that study delineated the principal moisture source regions, our approach provides a direct and globally comparable quantification of their relative contributions. Substantial regional variability also emerges (Supplementary Figs. 3–4): continental contributions are elevated in Scandinavia and central Europe, whereas the Iberian Peninsula exhibits tropical contributions reaching up to 29% on average.

### Global contrasts in atmospheric river precipitation origin

**Fig. 4:**
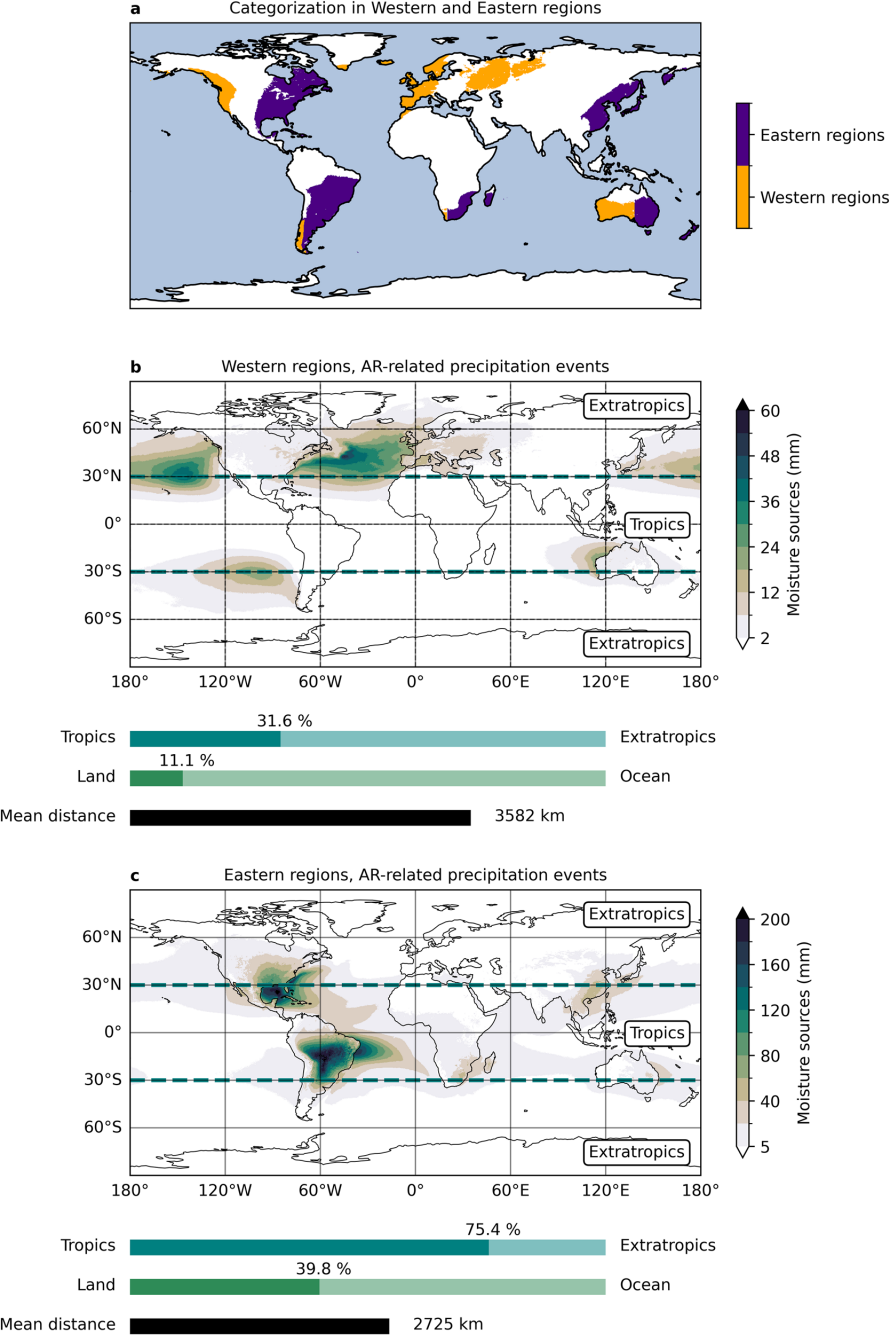
Global precipitation sources in ARs. **a** Categorization of the regions with an average precipitation attributable to ARs larger than 10 mm and fraction higher than 5% into eastern and western regions. Mean annual accumulated precipitation sources (color maps) and associated metrics (bars): tropical and continental contributions, and mean source-sink distance, for AR-related precipitation events affecting western (**b**) and eastern (**c**) regions.

When extending our analysis to the global scale, we identify both coherent patterns and pronounced contrasts among regions (Fig. 4). In western South America, the moisture source pattern closely mirrors that of western North America across the equator, with dominant tropical contributions and a secondary, weaker continental component (Supplementary Fig. 5). The mean source-sink distance is slightly larger than in North America, likely reflecting that the South American sink region is located farther from opposing continental margins across the ocean basin than in the North American case, allowing for longer uninterrupted oceanic trajectories. In contrast, eastern continental regions—including eastern North and South America, eastern South Africa, and Oceania (Supplementary Figs. 6–9)—show substantially smaller differences between AR and non-AR precipitation, particularly in the mean transport distance. In these regions, tropical contributions are generally higher due to proximity to the Tropics, while continental contributions also increase, averaging 52% in eastern South America, where the SALLJ incorporates large amounts of moisture evaporated from the Amazon into ARs affecting the region. East Asia represents an intermediate regime (Supplementary Fig. 10), characterized by enhanced tropical and continental contributions but still retaining discernible contrasts between AR and non-AR events.

To isolate robust global-scale behavior beyond regional variability, we classified AR-related precipitation events into western and eastern groups, based on the consistent similarities observed in moisture source fields over western North and South America and western Europe relative to other regions. Although previous studies have documented short-range continental recycling in specific eastern basins and long-range moisture transport in some western AR landfall regions^14,43–45^, these contrasts have not been systematically evaluated within a unified global framework. As shown in Fig. 4a, events affecting other western portions of the continents—such as South Africa, western Australia, Greenland, Iceland or even eastern Europe—are also included in the western category. The mean annual moisture source field for western regions (Fig. 4b) indicates an average tropical contribution of 31.6% across 33582 events, closely aligned with values for western North and South America and higher than the lower bound observed in Europe, together with a continental contribution of 11.1%. The relatively elevated continental contribution reflects the inclusion of Greenland and Iceland (Supplementary Fig. 11), as well as eastern Europe and western Russia, where moisture of continental origin is substantial. The mean source-sink distance is 3582 km, comparable to that of western North America and western Europe (Fig. 3). In contrast, eastern regions are dominated by eastern North and South America (Fig. 4c) and display significantly larger tropical and continental contributions (75.4% and 39.8%, respectively, across 77897 events), together with a shorter mean transport distance of 2725 km.

**Fig. 5:**
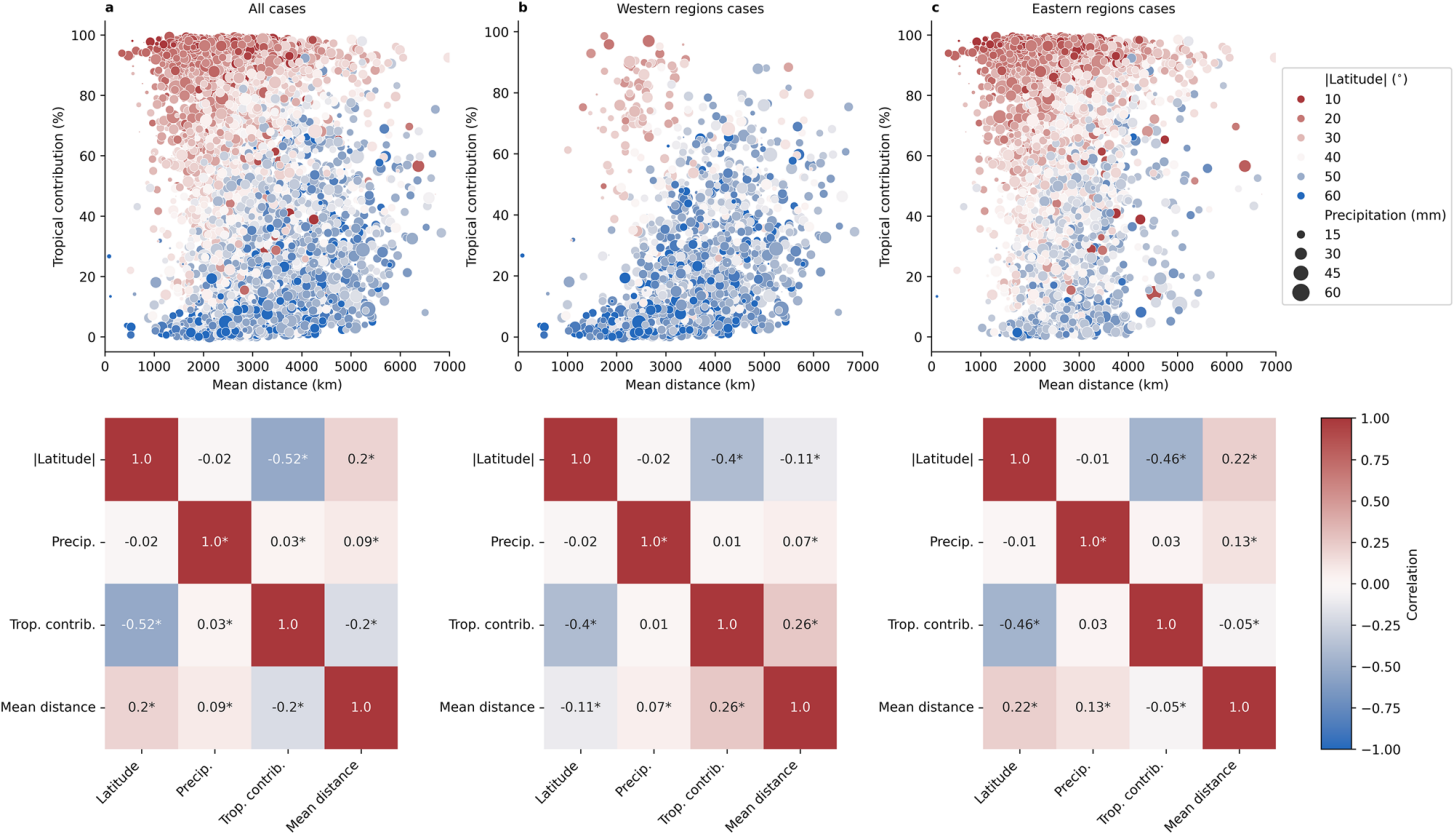
Phase space of AR moisture source features. At the top, scatter plot of tropical contribution versus mean source-sink distance for 5000 randomly selected AR-related precipitation events, where dot color indicates the latitude of the event, and dot size represents the amount of precipitation. At the bottom, Pearson correlation matrix for the four variables, with asterisks indicating significant correlations (*p* = 0.025). Panels **a**–**c** correspond to the whole sample, western regions, and eastern regions, respectively.

Figure 5 shows that the western–eastern categorization is inherently embedded in the global results and naturally emerges in a well defined phase space framework. The scatter of 5000 randomly selected AR-related precipitation events (Fig. 5a–c), shown in terms of mean source-sink distance versus tropical contribution and separated by all, western, and eastern cases, reveals a clear dynamical organization. The upper-left corner of this phase space—characterized by lower latitudes, shorter transport distances and high tropical contributions—is dominated by eastern-region events. In contrast, the lower-right corner—corresponding to higher latitudes, longer transport distances and weaker tropical influence—is primarily occupied by western-region events. This structure highlights a gradual transition from tropical-dominated, short-range moisture transport to extratropical, long-range transport regimes, consistent with the distinct dynamical environments governing AR evolution in the eastern and western sectors.

The Pearson correlation matrices (bottom panels of Fig. 5) further clarify the physical meaning of this phase space organization. In all cases, the strongest and most systematic relationship is the negative correlation between latitude (in absolute value) and tropical contribution, an expected but fundamental constraint reflecting the systematic decrease in tropical contribution as ARs evolve poleward away from their tropical formation region. For western events, a positive correlation emerges between tropical contribution and source-sink distance, indicating that ARs with stronger tropical influence tend to involve longer-range moisture transport. This feature is absent for eastern events, for which enhanced tropical contribution does not imply longer transport. Instead, eastern regions exhibit a positive correlation between latitude and transport distance, consistent with the notion that low-latitude ARs represent an earlier developmental phase in which the system already fulfills the AR criteria but tropical moisture export is still local, whereas longer transport distances are achieved as systems migrate poleward. Eastern events also show a positive correlation between precipitation and mean distance, indicating that the most intense rainfall does not occur during the low-latitude, incipient phase of AR development, but rather as the system elongates and dynamical forcing intensifies under stronger mid-latitude winds. Notably, in no region does precipitation display a robust direct correlation with either latitude or tropical contribution alone: ARs reaching higher latitudes, despite the reduced share of tropical moisture content, can still generate comparable precipitation amounts. This reflects the progressive replacement of tropical moisture by extratropical oceanic and continental sources and, importantly, the increasing dynamical efficiency of poleward-moving systems. ARs reaching higher latitudes are more likely to interact with extratropical cyclones and incorporate structures such as warm conveyor belts^46,47^, which are among the primary precipitation-generating flows in these cyclones. Additionally, the relative orientation between the moisture flux and the local topography can have a greater influence on precipitation than the absolute amount of water transported by the AR^48^, helping to explain the particularly large amounts of AR-related precipitation observed in high-latitude regions with steep coastal terrain, such as Norway and Greenland.

## Discussion

This study provides a global, climatologically robust quantification of the moisture sources feeding AR–related precipitation and reveals a coherent large-scale organization of moisture transport that is consistent across regional-scale differences. Although the balance between tropical and extratropical, oceanic and continental, or local and remote moisture sources varies widely across the globe, this diversity is organized into two fundamentally distinct regimes. Western continental regions are characterized by moisture transport dominated by extratropical and oceanic sources and by long-range pathways, whereas eastern continental regions display a stronger influence of tropical and continental moisture and comparatively shorter transport distances. By combining a global perspective with a long-term quantitative framework, our results move beyond the regional scope of most previous studies and establish a unified view of moisture origins for precipitation in ARs at the planetary scale.

**Fig. 6:**
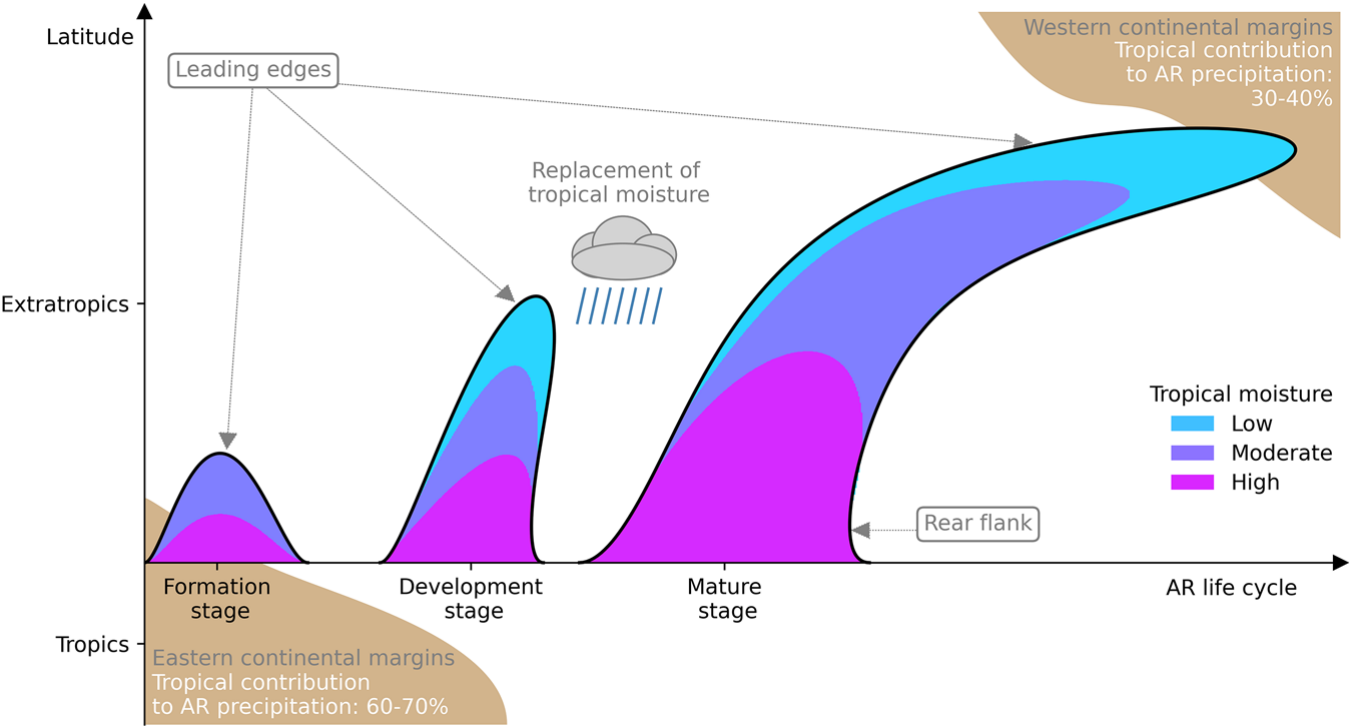
Schematic of the AR life cycle and moisture transport. Evolution of an AR from formation in the Tropics near eastern continental margins to maturity and landfall over western continental regions. Color shading represents the amount of tropical moisture, and typical tropical contributions to AR-related precipitation are indicated on the eastern and western continents.

The emergence of this western–eastern contrast is not merely geographical but reflects intrinsically different moisture transport dynamics embedded in the AR life cycle. Supplementary Figs. 15–24 show that, in western regions, AR-related precipitation consistently draws more moisture from tropical and oceanic sources than non-AR precipitation, while in eastern regions, tropical and continental contributions, as well as transport distances, exhibit greater interregional variability and differ less systematically between AR and non-AR events. Dynamically, these contrasts are consistent with the typical environments in which ARs develop and make landfall (see Fig. 6 for a schematic and Supplementary Fig. 26 for a real case). Western margins, such as western North America and Europe, are predominantly affected by mature ARs that have undergone prolonged oceanic transport before landfall, explaining the larger source-sink distances identified here. These ARs are typically located ahead of the cold front of extratropical cyclones^3,19^, within areas of strong moisture flux and low-level convergence along the filament. In contrast, eastern regions are more often influenced by the rear flank of mature ARs or the leading edge of developing ones, where local tropical moisture and convective activity play a more prominent role. Enhanced continental evaporation and secondary recycling can contribute to the higher terrestrial contribution identified in our results. Moreover, some eastern basins (e.g., East Asia and eastern South America) are located within monsoon-influenced domains^14,49^. In East Asia, the connectivity between ARs and monsoon flows remains under debate^50^. Our results nevertheless suggest that ARs are an important feature of the climate of East China, Korea, and Japan (see Supplementary Fig. 10), where they are closely linked to monsoonal rainfall^51^, and are frequently characterized by persistent southwesterly low-level jets during the monsoon season, contributing to a moisture transport regime that differs structurally from that of western continental margins. It should be noted, however, that not all western events are dynamically connected to preceding eastern ARs. This means that ARs heading towards western continental margins often originate in the middle of the ocean, with no land impacted by their incipient stages or rear flank, or that ARs from eastern continental regions frequently do not make it all the way across an ocean basin and dissipate somewhere over the open sea instead. The proposed categorization should be interpreted as a statistical and climatological pattern grounded in dynamical and physical processes, rather than a deterministic description of individual AR life cycles.

The distinct correlation patterns between latitude, transport distance and tropical contribution further support this dynamical interpretation. The strong replenishment of moisture along AR pathways, inferred from the anticorrelation between latitude and tropical contribution, does not imply a vanishing tropical influence at higher latitudes^25^; rather, it points to a predominantly indirect role. Moisture exported from the tropics is critical during the initial stage of AR formation, preconditioning the coherent filament that later enables efficient poleward moisture transport. This initial filament is typically organized by low-level flow along the equatorward flank of subtropical high-pressure systems, often in association with low-level jets (see Supplementary Fig. 26 for a real case). In some cases, tropical disturbances embedded in this flow can generate precursor moisture plumes and contribute to the initial organization of the system before transitioning into the mid-latitudes. Subsequent interaction with an extratropical cyclone enhances convergence along the filament, facilitating the incorporation of non-tropical sources—which ultimately dominates precipitation at higher latitudes—and altering the moisture composition prior to landfall. At the same time, the latent heat released as tropical moisture precipitates in mid-latitudes is more directly conveyed toward higher latitudes than the water itself, indicating a proportionally greater tropical heat delivery. This divergence between water supply and heat transport may explain why high-latitude AR impacts are often thermodynamically modulated by tropical forcing, despite limited direct tropical moisture input.

These results are nevertheless subject to some limitations. The variability in AR frequency shown in Fig. 1 indicates that uncertainties related to the detection algorithms may be non-negligible. To assess the robustness of our conclusions, we recomputed the key metrics characterizing moisture origins for an independent year using eight AR detection algorithms, namely the seven global ARTMIP Tier 2 algorithms and CG-Climate (Supplementary Fig. 12). The spread across algorithms reveals no systematic bias, with our reference method consistently lying within the interquartile range. Importantly, the qualitative separation between western and eastern regimes is preserved across all detection methods with median tropical contributions below 40% for western cases and above 60% for eastern cases, and with western regions systematically exhibiting lower continental contributions and longer transport distances than eastern regions. This demonstrates that the main conclusions of this study are robust to uncertainties associated with AR detection and are not dependent on a specific identification and moisture tracking framework.

The intensity of the AR-related precipitation may also influence the resulting moisture source assessment. In this study, we only evaluated precipitation events surpassing the threshold of 10 mm per day, to balance computational efficiency and statistical significance (see Methods). To assess sensitivity to this choice, we recomputed the main metrics for an independent year using different rainfall thresholds (5, 10, 20, and 30 mm/day; see Supplementary Fig. 13). Tropical contributions increase slightly with precipitation intensity in most regions, in the order of 5% (Supplementary Fig. 14). This difference is small and would in no case alter the main conclusions of our study. Our results are therefore robust with respect to this threshold choice. Moreover, in a previous study we showed that our Lagrangian framework tends to slightly underestimate tropical contributions for western regions^29^, also on the order of 0–5%. Taking these discrepancies into account, it is reasonable to suggest that the best estimate for the mean tropical contribution along western coasts likely lies in the range of 30–40%, that is, slightly higher than the value of 31.6% reported in the Results (Fig. 4).

Viewed in a global context, the emergence of two contrasting moisture transport regimes underscores that ARs cannot be treated as a single dynamical entity worldwide, but rather as systems whose moisture supply is shaped jointly by their stage of development and their geographical setting, similarly to the broad category of warm conveyor belts of extratropical cyclones to which they mostly belong^52^. This distinction provides a physically grounded framework for interpreting regional contrasts in AR-related precipitation and its variability. More broadly, the global organization of AR moisture sources identified here clarifies how the balance between tropical and extratropical moisture contributions is structured at the planetary scale and may help to understand how this balance could shift under a changing climate.

## Methods

### AR detection and associated precipitation

To detect ARs, we use a deep learning algorithm capable of segmenting both ARs and tropical cyclones (TCs) in simulated climate data^33,34^. The algorithm, known as CG-Climate, is based on a convolutional neural network adapted from a Context Guided Network^53^ (CGNet), a lightweight architecture designed for semantic segmentation. The model was trained on ClimateNet^41^, an expert-annotated dataset derived from simulations with the Community Atmospheric Model^54^ (CAM5.1) at 25 km resolution. ClimateNet includes integrated water vapor, integrated vapor transport, vorticity, surface winds, 850 hPa winds, and sea level pressure, together with expert labels on these fields for 1000 time steps. For training CG-Climate, only integrated water vapor, 850 hPa winds, and sea level pressure were used, along with the expert-provided labels^34^. We use a PyTorch implementation of the model (https://github.com/andregraubner/ClimateNet, last access: 7 January 2026) with ERA5 reanalysis data as input^39^. After obtaining the AR labels and to reduce false positives, we impose additional geometric requirements: detected objects must be at least 2000 km in length, less than 600 km in width, and not be confined entirely within 25° S and 25° N.

ARs are detected on an hourly basis, after which we identify precipitation events associated with them (AR-related precipitation events). Specifically, for each 6-hour interval and each grid cell with precipitation, we attribute that amount of rainfall to an AR if, during the same interval or the previous one, at least one AR detection occurred at that location. We repeat this procedure for the whole day and aggregate the results to obtain daily AR-related precipitation. Finally, we retain only areas exceeding 10 mm per day and apply a spatial clustering algorithm—specifically, a connected component labelling method included in SciPy^55^—to obtain a database of daily AR-related precipitation events.

For precipitation events not associated with ARs, the same aggregation and clustering algorithms are applied to the field obtained by subtracting AR-related precipitation from total precipitation. Given the large number of resulting events, only regions where AR precipitation is significant are analyzed. Moreover, instead of computing moisture sources for all non-AR precipitation events, a representative sample is selected by dividing the globe into 496 equal-area boxes and randomly selecting up to 20 events per year and box. To avoid biases in the resulting annual moisture source fields, we compute the precipitation bias introduced by this sampling and use it to rescale the contribution of each event before accumulating them into the annual fields.

### Lagrangian moisture tracking

The moisture tracking methodology employed to obtain precipitation sources for each rainfall event is based on the Lagrangian particle dispersion model FLEXPART v10.4^35^, which has been widely applied to studies of the origin and transport of atmospheric humidity^21,56,57^. FLEXPART integrates the trajectory equation^58^:

1 $$\frac{d{\boldsymbol{X}}}{{dt}}={\boldsymbol{v}}\left[{\boldsymbol{X}}\left(t\right)\right],{\boldsymbol{v}}=\bar{{\boldsymbol{v}}}+{{\boldsymbol{v}}}_{{\boldsymbol{t}}}+{{\boldsymbol{v}}}_{{\boldsymbol{m}}},$$

where v is the total wind velocity, composed of a grid-scale component $\bar{v}$, and additional terms representing turbulent (v_t_) and mesoscale (v_m_) wind fluctuations. The grid-scale wind is obtained from ERA5 reanalysis data^39^, while the subgrid-scale fluctuations are parameterized in FLEXPART as stochastic Markov processes^59^.

In this study, we conducted a global simulation with 10 million air parcels spanning the period 1980-2023. Parcels are initialized using the domain-filling option in FLEXPART, with equal air mass assigned to each parcel, a vertical distribution proportional to atmospheric density, and a horizontally uniform distribution over the global model domain. They are then transported forward in time by the mean, turbulent, and mesoscale wind components, following Eq. (1). Among other atmospheric variables, FLEXPART provides the three-dimensional position, specific humidity, pressure, density and temperature of each parcel, along with the height of the atmospheric boundary layer, at a 3-hourly temporal resolution and over the entire simulation period.

To obtain precipitation sources, we select the 30 days backward trajectories for each rainfall event and apply our own implementation of the Dirmeyer and Brubaker (1999) methodology^36^, hereafter DB99. This diagnostic is widely used in Lagrangian moisture tracking models such as UTrack^60^ and BTrIMS^61^. In addition to FLEXPART output, it requires ERA5 3-hourly evaporation and integrated water vapor fields at 0.25° resolution. DB99 distributes precipitation in each grid cell among all parcels above it proportionally to their specific humidity, and then allocates each parcel’s share of rainfall to the grid cells beneath it along its 30-day backward trajectory. To do so, at each 3-hourly time step a fraction of this share—equal to the ratio of evaporation to integrated water vapor—is allocated to the underlying grid cell, and the parcel share is updated accordingly, i.e. the amount of rainfall it represents is reduced by the same factor. This process continues backward in time, yielding a spatial distribution of moisture sources for each parcel’s initial share of rainfall. Finally, the distributions from all parcels and all grid cells are aggregated to produce the two-dimensional precipitation source field for the event, from which the key metrics analyzed here—tropical and continental contributions, and mean source–sink distance—are computed. The tropical contribution is defined as the fraction of moisture originating between 30°S and 30°N, while the mean source–sink distance corresponds to the average distance between each moisture source pixel and the geometric center of the sink, weighted by the amount of moisture contributed by that pixel.

While the original DB99 method considers all parcels released at the time and location of the rainfall episode—weighted vertically by their specific humidity and horizontally by the precipitation rate—we exclude from the calculation parcels released below 2 km altitude. This modification is based on the rationale that such parcels typically do not contribute directly to precipitation at that time, as they are often located below the active cloud layer. This modified DB99 framework improves the representation of remote moisture contributions and has been shown to be more consistent with the WRF with Water Vapor Tracers model—considered one of the most accurate moisture tracking methodologies—specifically for AR precipitation events^29^.

## Supplementary information

Supplementary figures

## Data Availability

ERA5 data on single levels used to detect ARs and compute moisture sources were downloaded from the Climate Data Store, https://cds.climate.copernicus.eu/datasets/reanalysis-era5-single-levels (last access: 7 January 2026). FLEXPART simulations are available upon request.

## Code availability

FLEXPART v10.4 can be accessed through https://www.flexpart.eu/ (last access: 7 January 2026), and the CG-Climate model to detect ARs is available on GitHub (https://github.com/andregraubner/ClimateNet, last access: 7 January 2026). Finally, the implementation of the DB99 methodology to compute moisture sources and analysis scripts are published on GitHub (https://github.com/crespoalfredo/ARs_MoistureSources, last access: 7 Jan 2026).
