# Supplementary material for: Global asymmetries in the moisture origins of atmospheric river precipitation
Source: NPJ Clim Atmos Sci. 2026 Apr 8;9(1):137. doi: 10.1038/s41612-026-01408-6 (PMC13271881; doi:10.1038/s41612-026-01408-6)
Supplement: Supplementary file 1 — Supplementary figures [file 41612_2026_1408_MOESM1_ESM.pdf]

Supplementary figures

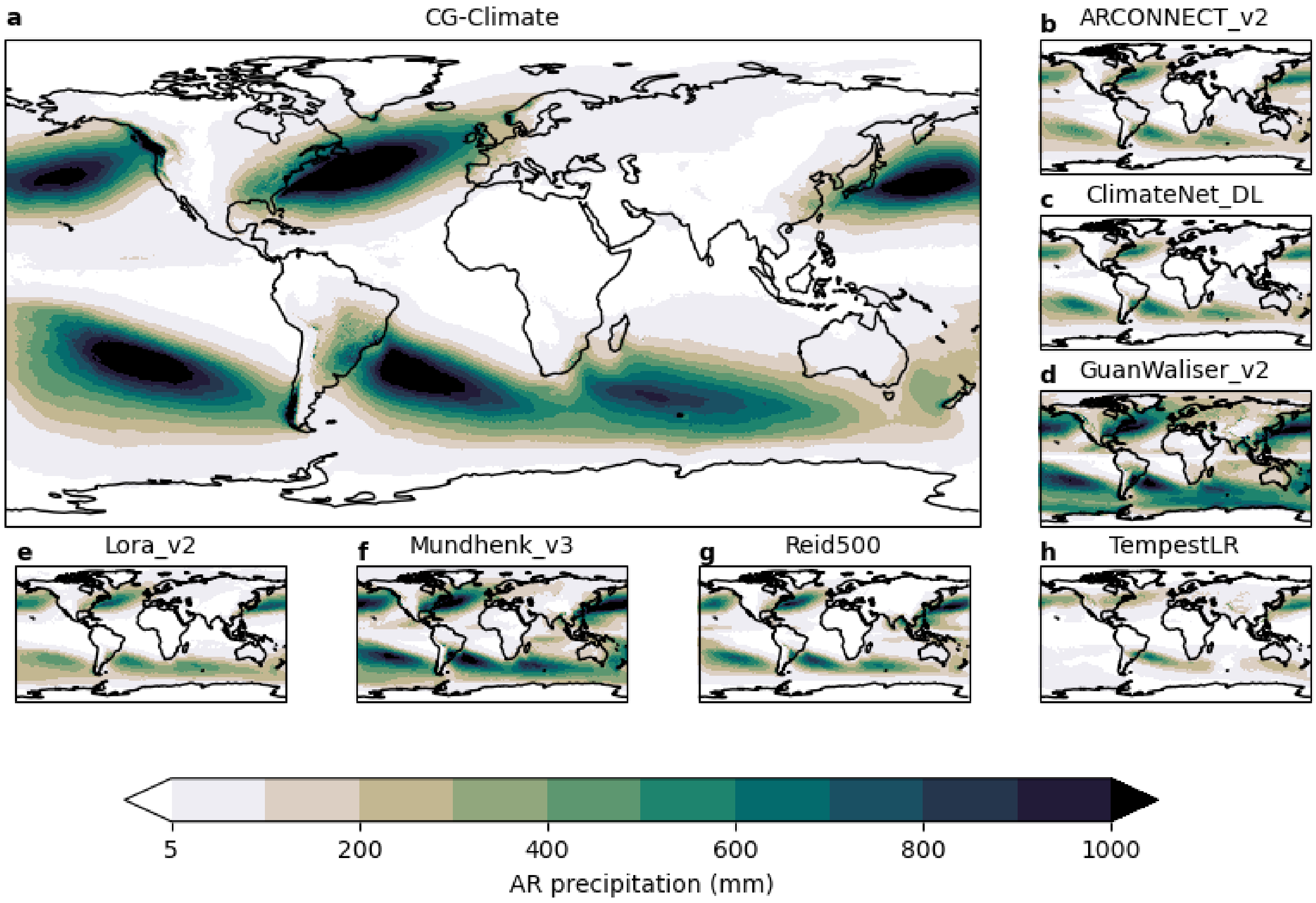

Supplementary Fig. 1 | AR precipitation. Average annual AR-related precipitation for the DL algorithm used in this study (CG-Climate, panel a) and for the rest of algorithms participating in the ARTMIP project Tier 2 with global coverage (b-h), during the period 2000-2019.

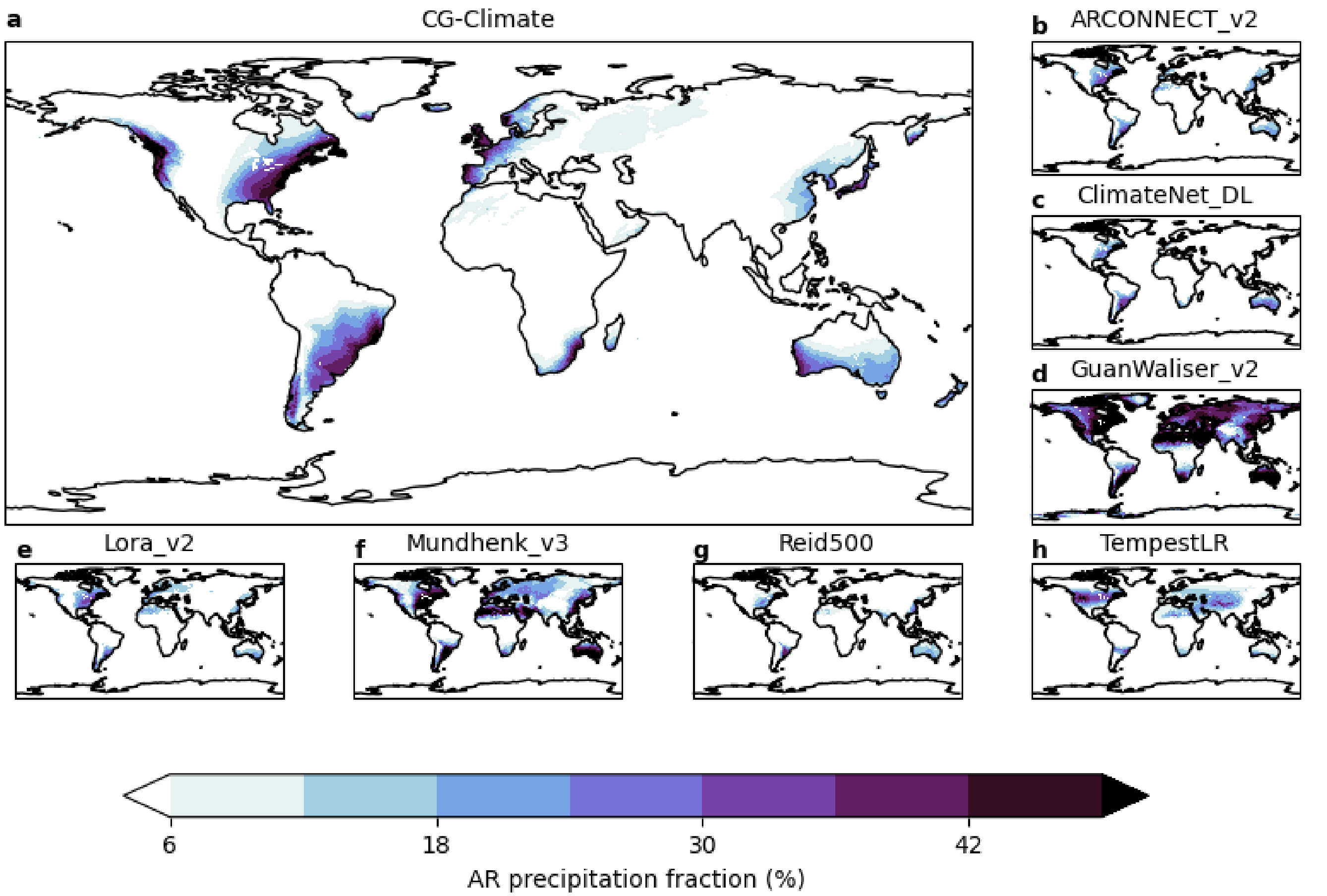

Supplementary Fig. 2 | AR precipitation fraction. Average annual fraction of precipitation associated with ARs for the DL algorithm used in this study (CG-Climate, panel a) and for the rest of algorithms participating in the ARTMIP project Tier 2 with global coverage (b-h), during the period 2000-2019.

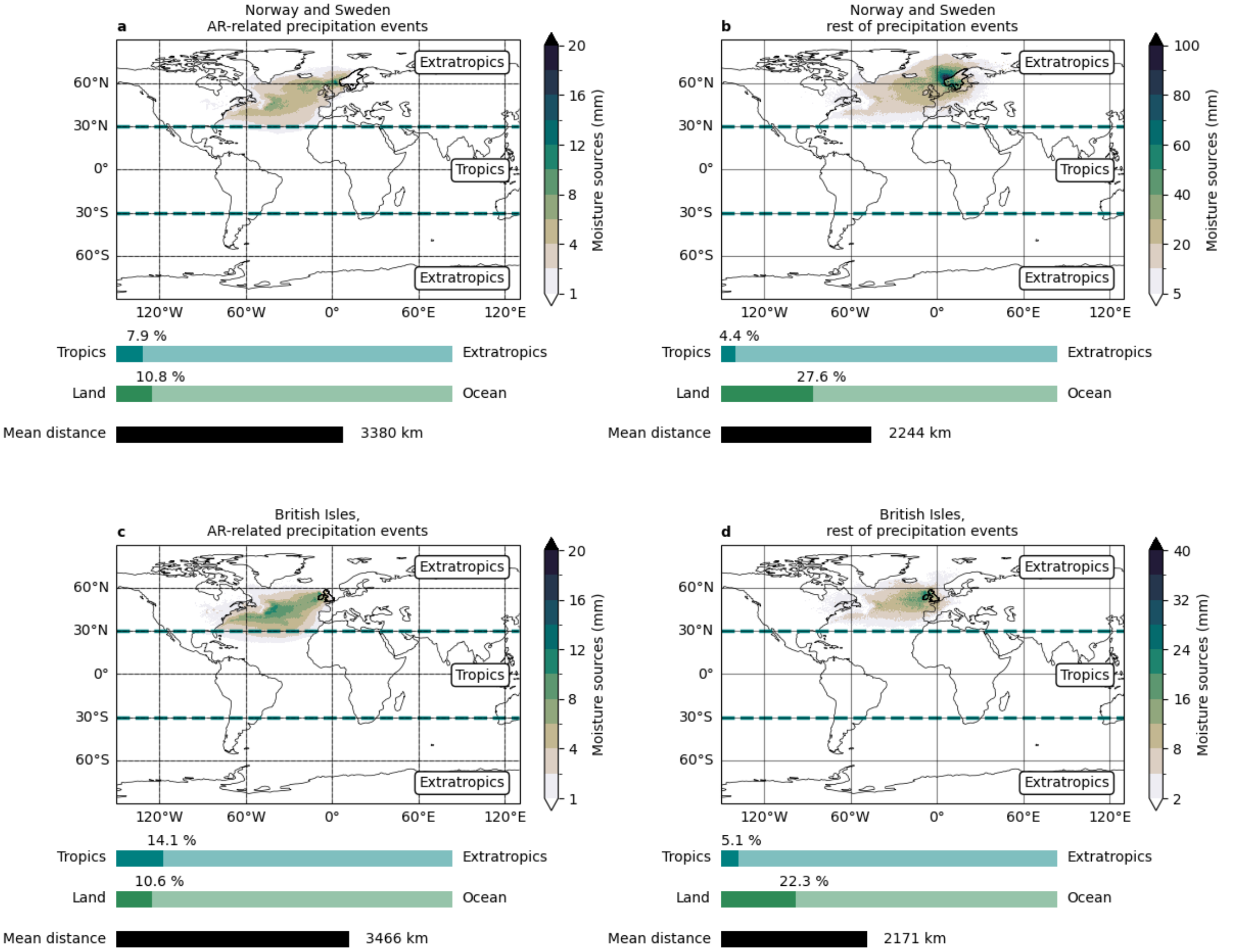

Supplementary Fig. 3 | Precipitation sources western Europe (1). Mean annual accumulated moisture sources, tropical and continental contributions, and mean distance from source to sink for precipitation events affecting Scandinavia (a and b) and the British Isles (c and d), during the period 1980-2023. Left panels (a and c) correspond to AR-related precipitation events, while right panels (b and d) correspond to the rest of the events.

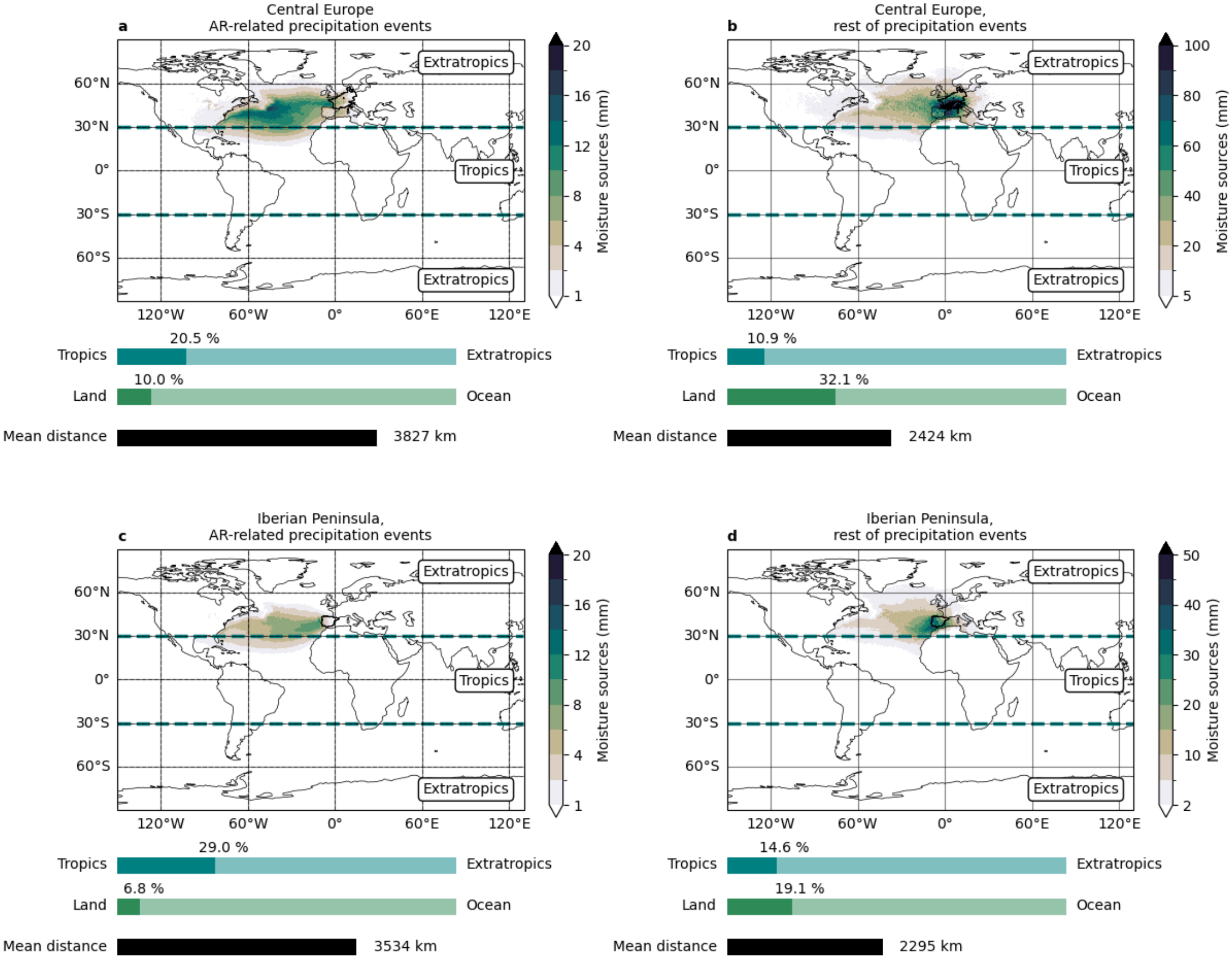

Supplementary Fig. 4 | Precipitation sources western Europe (2). Mean annual accumulated moisture sources, tropical and continental contributions, and mean distance from source to sink for precipitation events affecting central Europe (a and b) and the Iberian Peninsula (c and d), during the period 1980-2023. Left panels (a and c) correspond to AR-related precipitation events, while right panels (b and d) correspond to the rest of the events.

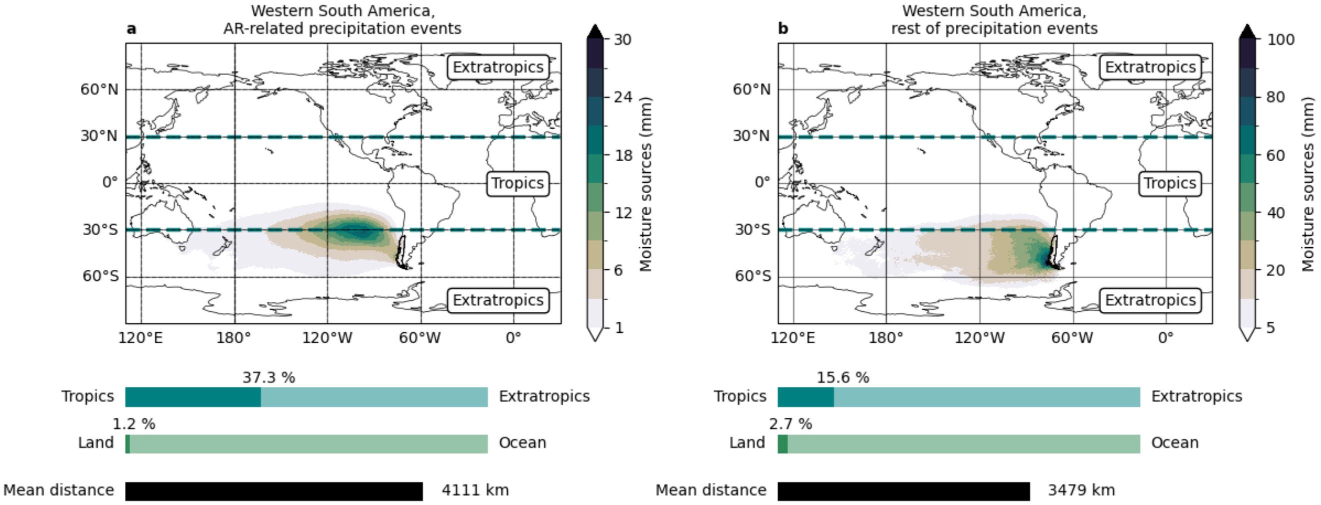

Supplementary Fig. 5 | Precipitation sources western South America. Mean annual accumulated moisture sources, tropical and continental contributions, and mean distance from source to sink for precipitation events affecting western South America, for AR-related precipitation events (a) and rest of events (b), during the period 1980-2023.

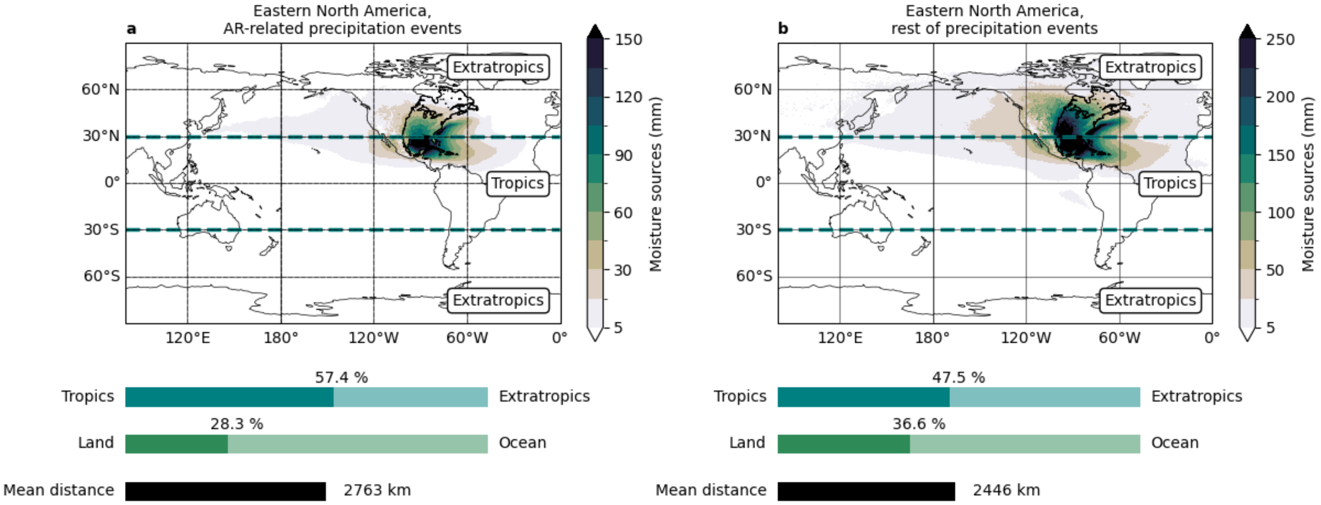

Supplementary Fig. 6 | Precipitation sources eastern North America. Mean annual accumulated moisture sources, tropical and continental contributions, and mean distance from source to sink for precipitation events affecting eastern North America, for AR-related precipitation events (a) and rest of events (b), during the period 1980-2023.

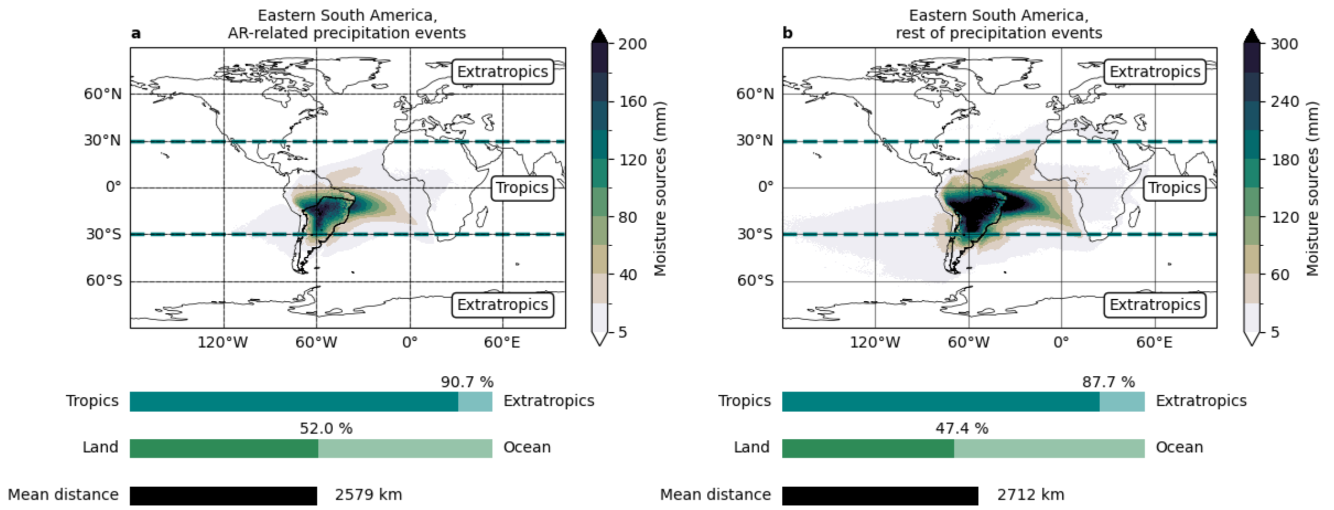

Supplementary Fig. 7 | Precipitation sources eastern South America. Mean annual accumulated moisture sources, tropical and continental contributions, and mean distance from source to sink for precipitation events affecting eastern South America, for AR-related precipitation events (a) and rest of events (b), during the period 1980-2023.

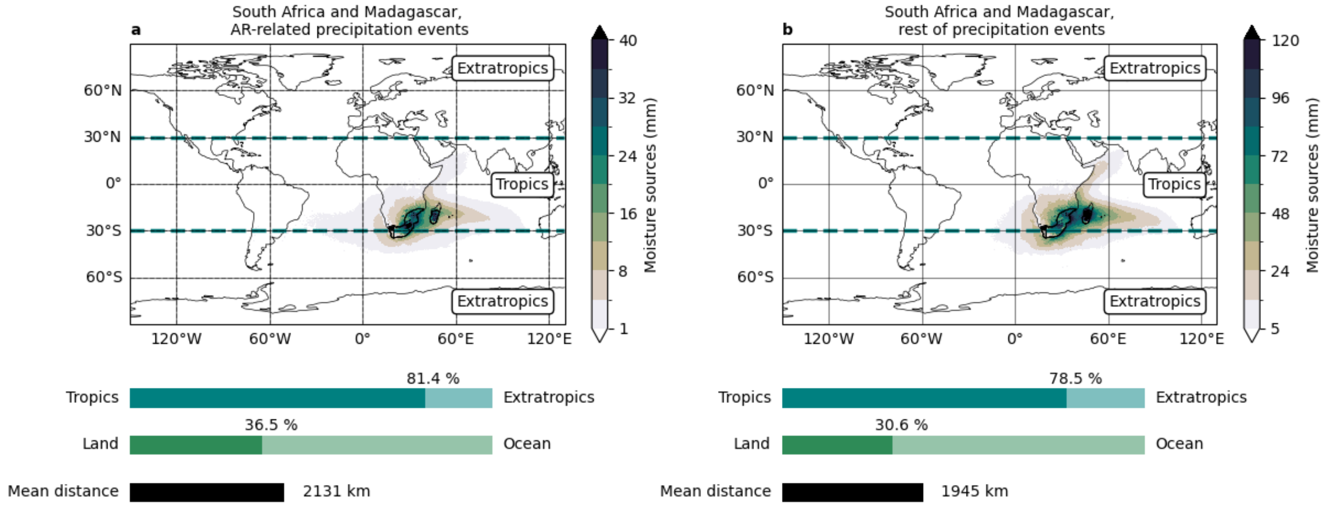

Supplementary Fig. 8 | Precipitation sources South Africa and Madagascar. Mean annual accumulated moisture sources, tropical and continental contributions, and mean distance from source to sink for precipitation events affecting South Africa and Madagascar, for AR-related precipitation events (a) and rest of events (b), during the period 1980-2023.

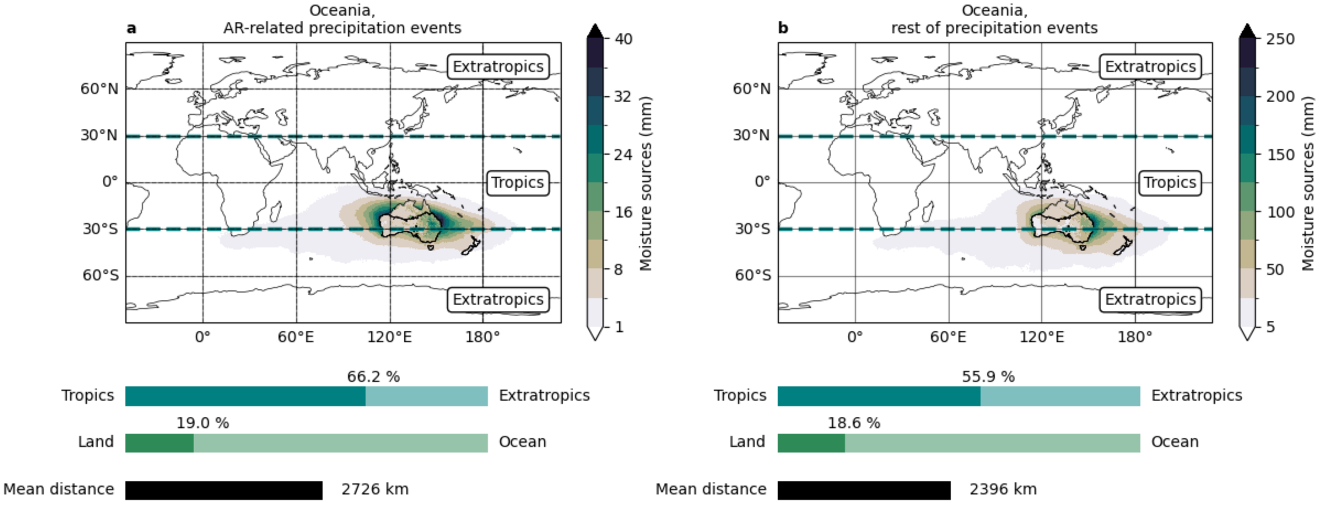

Supplementary Fig. 9 | Precipitation sources Oceania. Mean annual accumulated moisture sources, tropical and continental contributions, and mean distance from source to sink for precipitation events affecting Oceania, for AR-related precipitation events (a) and rest of events (b), during the period 1980-2023.

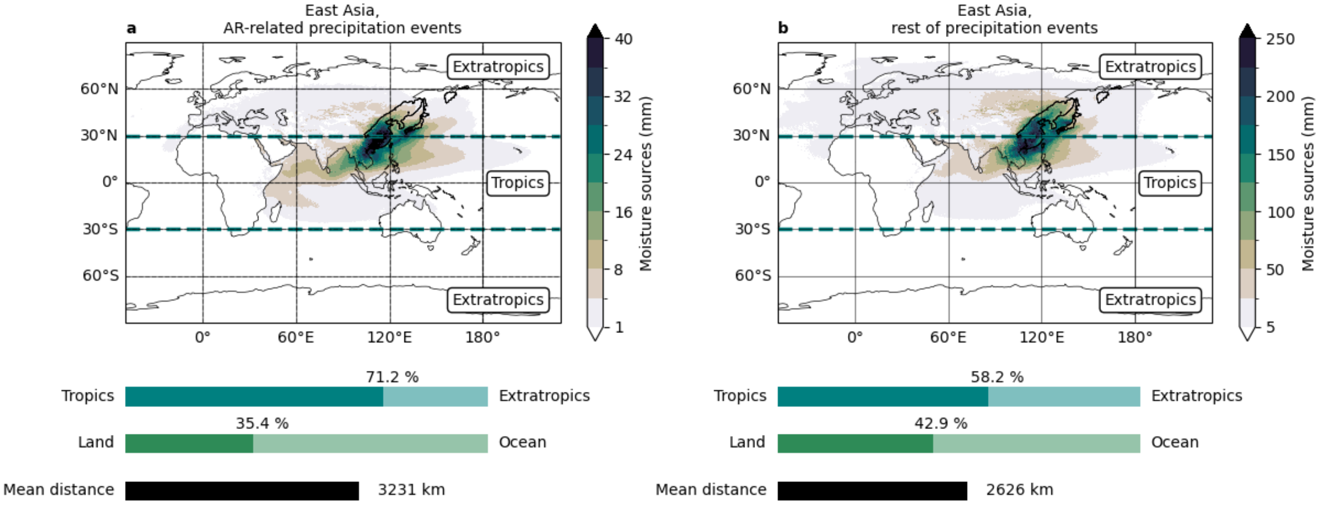

Supplementary Fig. 10 | Precipitation sources East Asia. Mean annual accumulated moisture sources, tropical and continental contributions, and mean distance from source to sink for precipitation events affecting East Asia, for AR-related precipitation events (a) and rest of events (b), during the period 1980-2023.

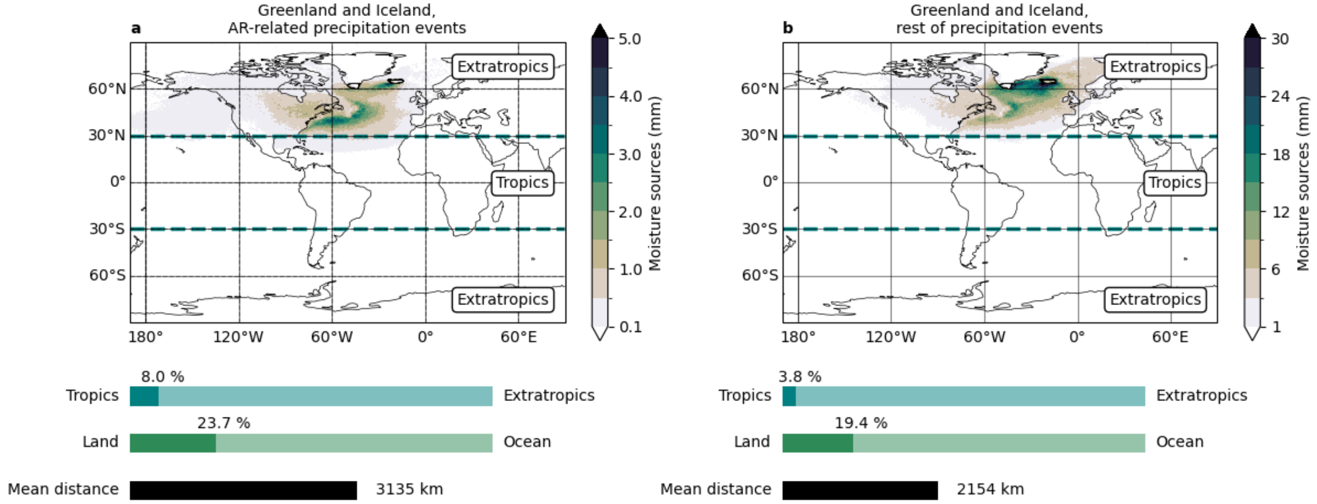

Supplementary Fig. 11 | Precipitation sources Greenland and Iceland. Mean annual accumulated moisture sources, tropical and continental contributions, and mean distance from source to sink for precipitation events affecting Greenland and Iceland, for AR-related precipitation events (a) and rest of events (b), during the period 1980-2023.

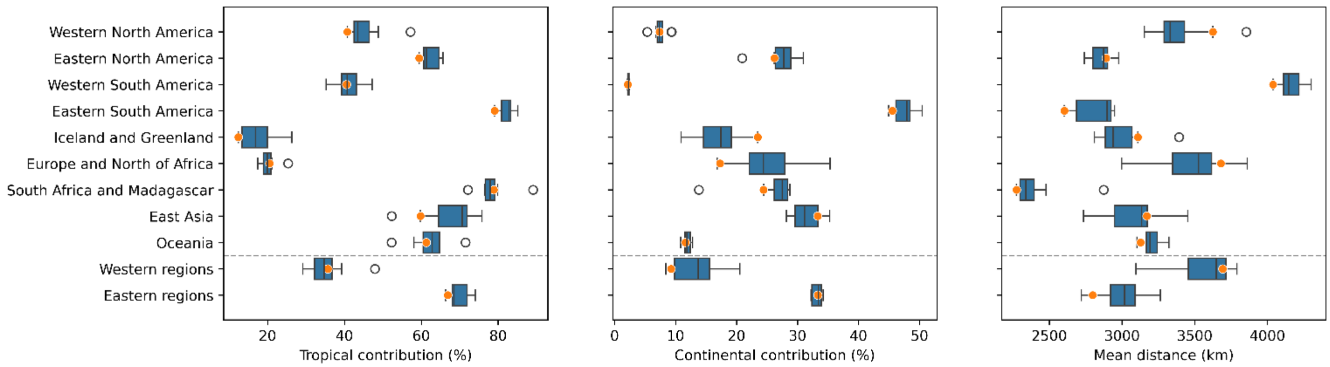

Supplementary Fig. 12 | Moisture source diagnostic with other algorithms. Tropical and continental contributions, and mean distance from source to sink, for selected regions affected by AR-related precipitation events in 2019. Boxplots represent the spread among eight global AR detection algorithms (seven ARTMIP Tier 2 methods plus CG-Climate), while orange dots indicate results from CG-Climate.

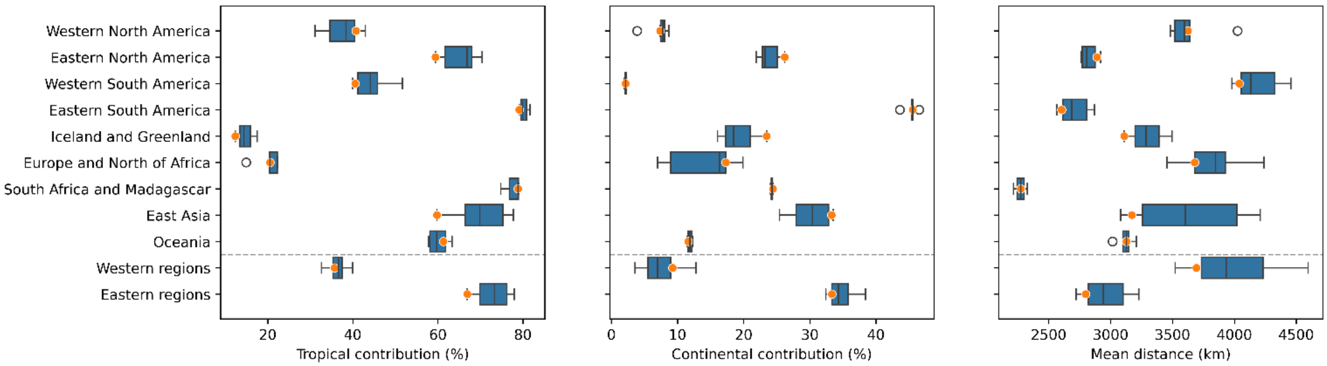

Supplementary Fig. 13 | Moisture source diagnostic with other thresholds (1). Tropical and continental contributions, and mean distance from source to sink, for selected regions affected by AR-related precipitation events in 2019. Boxplots represent the spread resulting from different thresholds for the identification of AR precipitation events (5, 10, 20, 30, 40 and 50 mm).

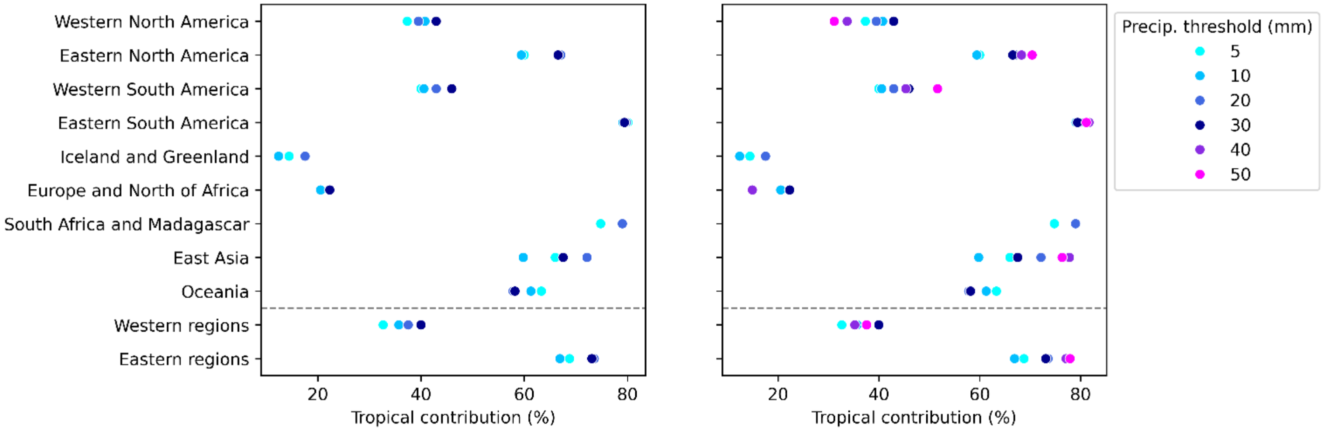

Supplementary Fig. 14 | Moisture source diagnostic with other thresholds (2). Tropical contributions for selected regions affected by AR-related precipitation events in 2019, where color indicates the chosen precipitation threshold to identify AR precipitation events. The left panel shows thresholds up to 30 mm, while the right panel also includes 40 and 50 mm.

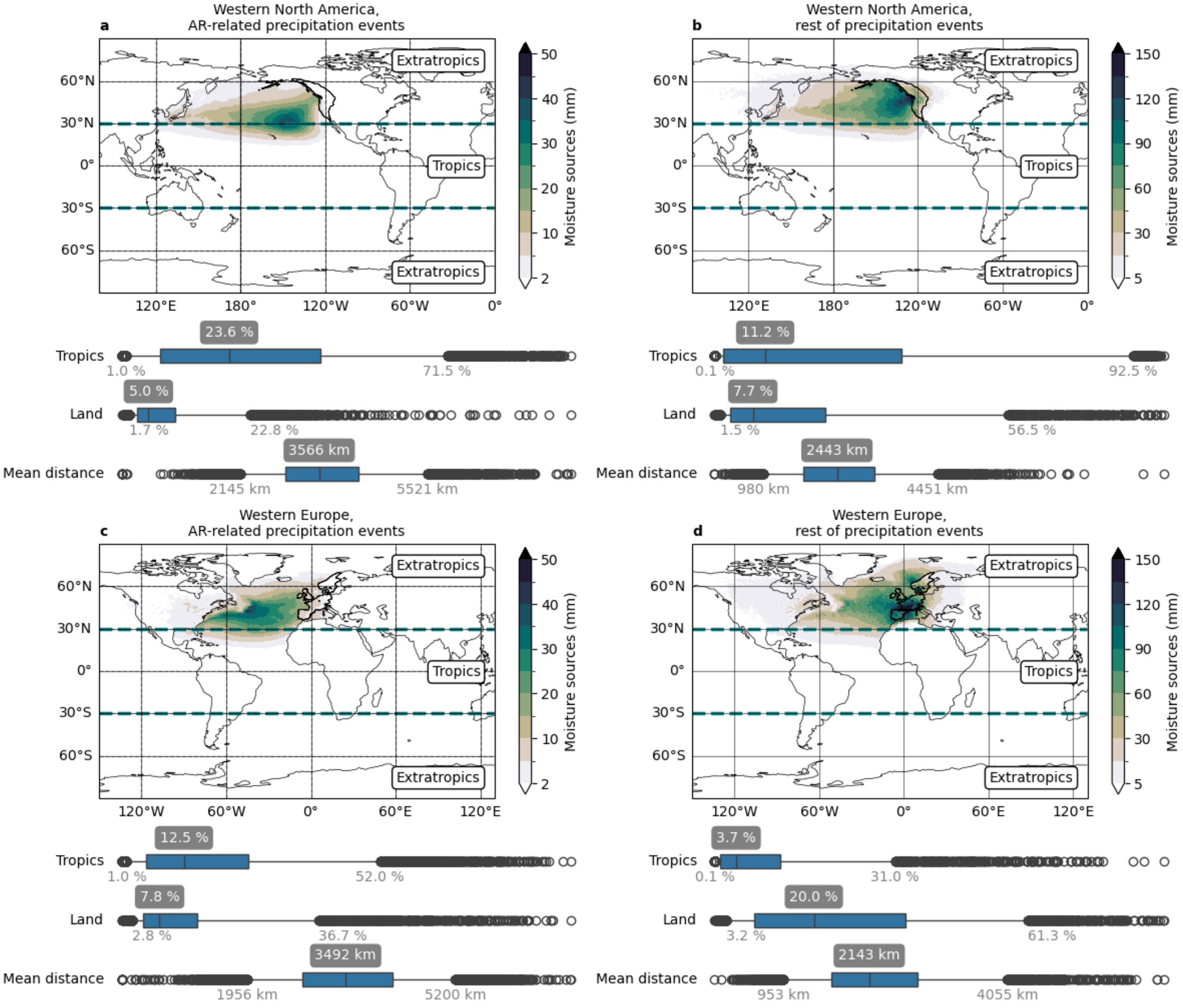

Supplementary Fig. 15 | Precipitation sources with boxplots AR hotspot regions. Mean annual accumulated moisture sources for precipitation events affecting western North America (a and b) and western Europe (c and d), during the period 1980-2023. Left panels (a and c) correspond to AR-related precipitation events, while right panels (b and d) correspond to the rest of the events. Boxplots display the event-level distributions of tropical and continental contributions, as well as the mean source-sink distance; dots indicate values outside the 5th-95th percentile range, and medians are marked.

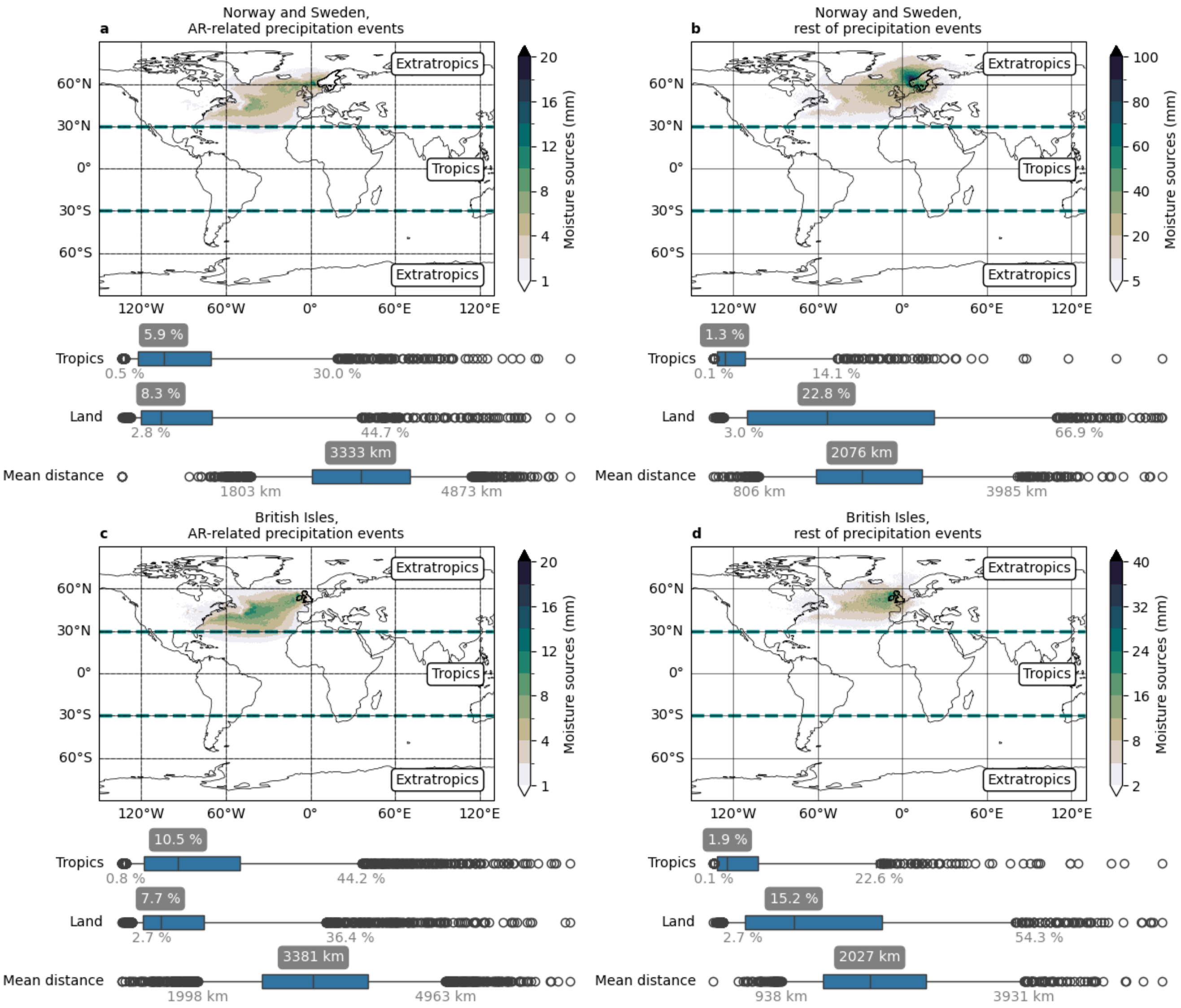

Supplementary Fig. 16 | Precipitation sources with boxplots western Europe (1). Mean annual accumulated moisture sources for precipitation events affecting western Scandinavia (a and b) and the British Isles (c and d), during the period 1980-2023. Left panels (a and c) correspond to AR-related precipitation events, while right panels (b and d) correspond to the rest of the events. Boxplots display the event-level distributions of tropical and continental contributions, as well as the mean source-sink distance; dots indicate values outside the 5th-95th percentile range, and medians are marked.

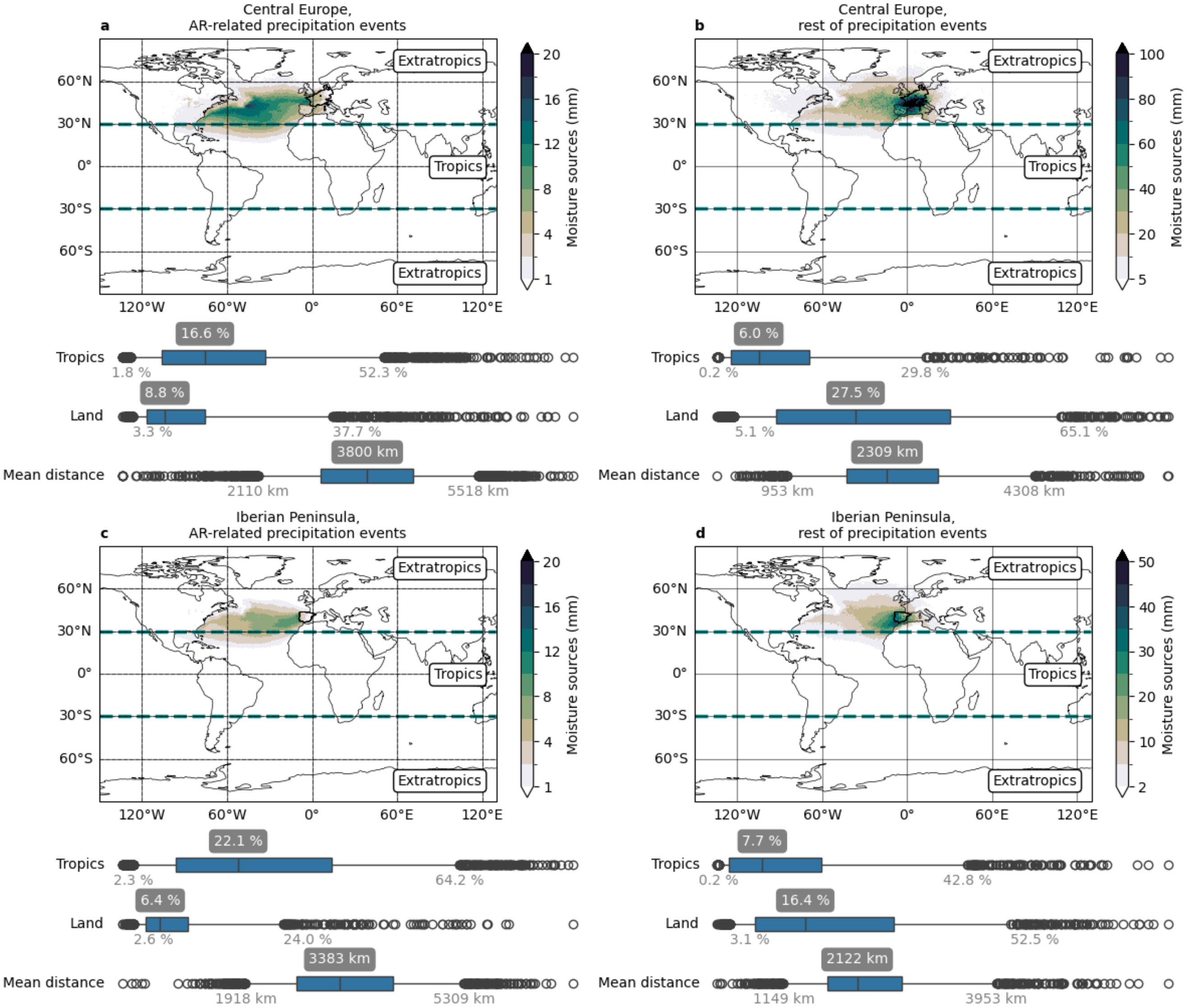

Supplementary Fig. 17 | Precipitation sources with boxplots western Europe (2). Mean annual accumulated moisture sources for precipitation events affecting central Europe (a and b) and the Iberian Peninsula (c and d), during the period 1980-2023. Left panels (a and c) correspond to AR-related precipitation events, while right panels (b and d) correspond to the rest of the events. Boxplots display the event-level distributions of tropical and continental contributions, as well as the mean source-sink distance; dots indicate values outside the 5th-95th percentile range, and medians are marked.

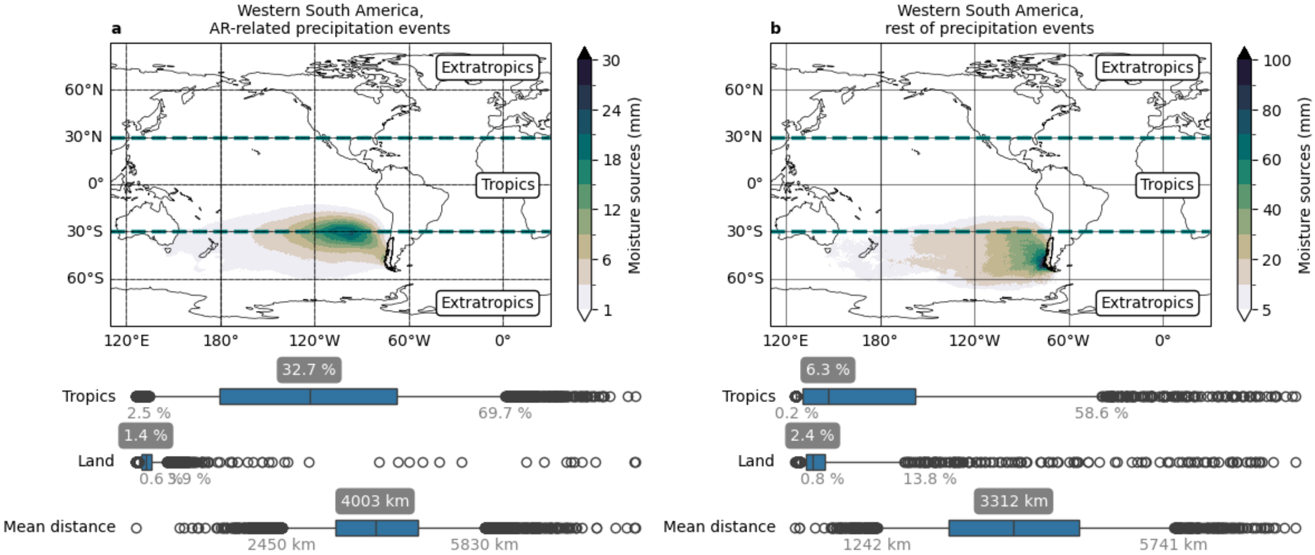

Supplementary Fig. 18 | Precipitation sources with boxplots western South America. Mean annual accumulated moisture sources for precipitation events affecting western South America, for AR-related precipitation events (a) and rest of events (b), during the period 1980-2023. Boxplots display the event-level distributions of tropical and continental contributions, as well as the mean source-sink distance; dots indicate values outside the 5th-95th percentile range, and medians are marked.

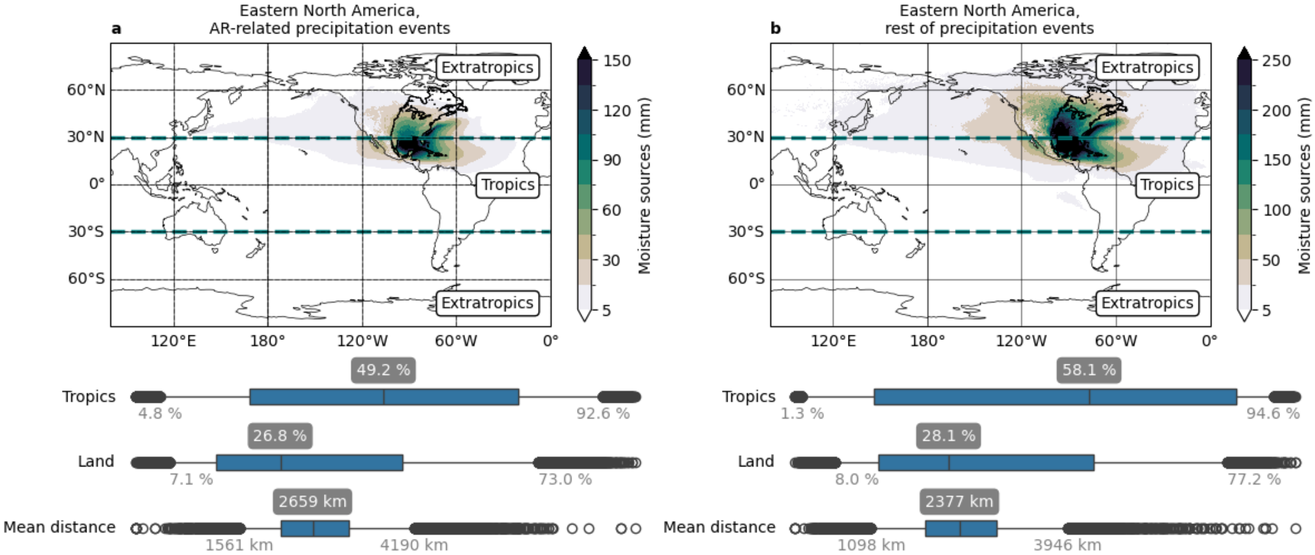

Supplementary Fig. 19 | Precipitation sources with boxplots eastern North America. Mean annual accumulated moisture sources for precipitation events affecting eastern North, for AR-related precipitation events (a) and rest of events

(b), during the period 1980-2023. Boxplots display the event-level distributions of tropical and continental contributions, as well as the mean source-sink distance; dots indicate values outside the 5th-95th percentile range, and medians are marked.

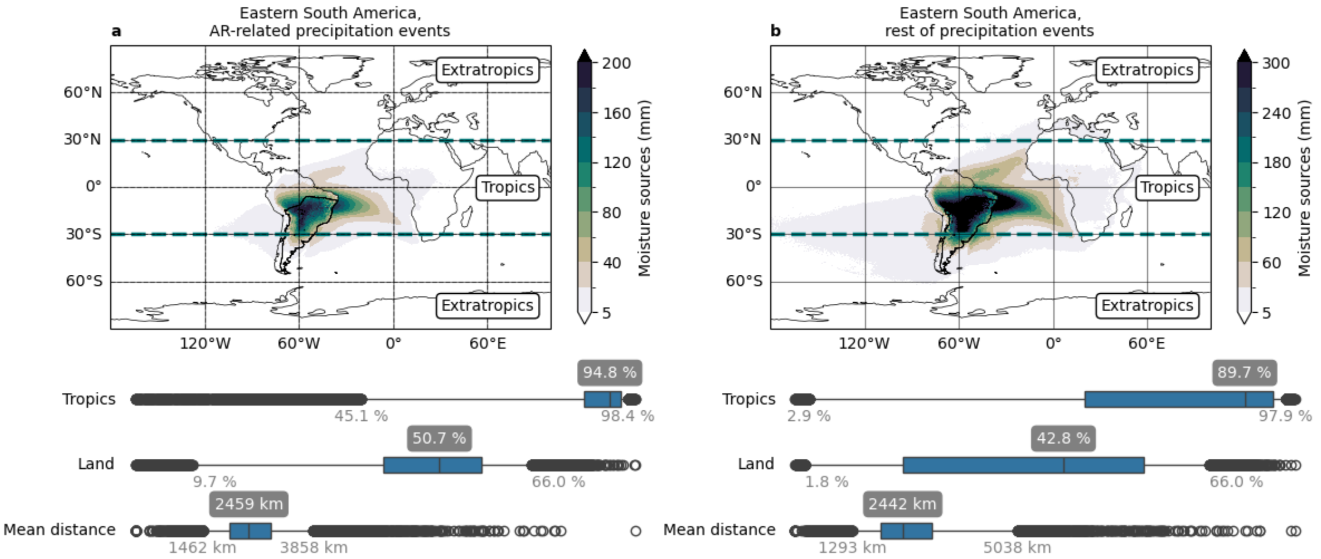

Supplementary Fig. 20 | Precipitation sources with boxplots eastern South America. Mean annual accumulated moisture sources for precipitation events affecting eastern South America, for AR-related precipitation events (a) and rest of events (b), during the period 1980-2023. Boxplots display the event-level distributions of tropical and continental contributions, as well as the mean source-sink distance; dots indicate values outside the 5th-95th percentile range, and medians are marked.

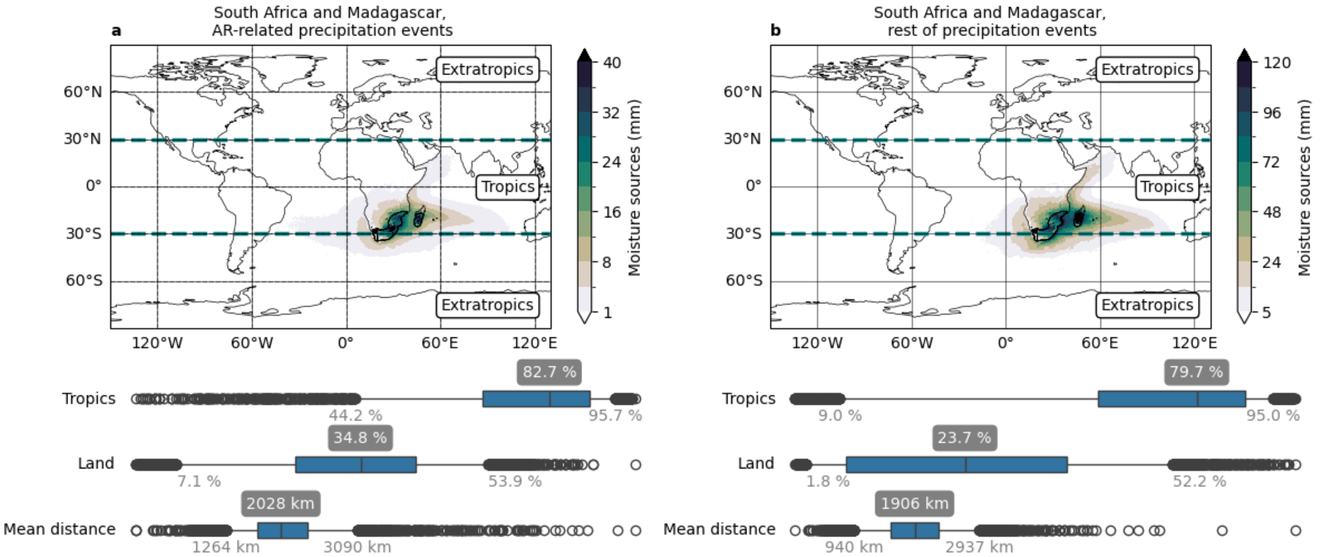

Supplementary Fig. 21 | Precipitation sources with boxplots South Africa and Madagascar. Mean annual accumulated moisture sources for precipitation events affecting South Africa and Madagascar, for AR-related precipitation events (a) and

rest of events (b), during the period 1980-2023. Boxplots display the event-level distributions of tropical and continental contributions, as well as the mean source-sink distance; dots indicate values outside the 5th-95th percentile range, and medians are marked.

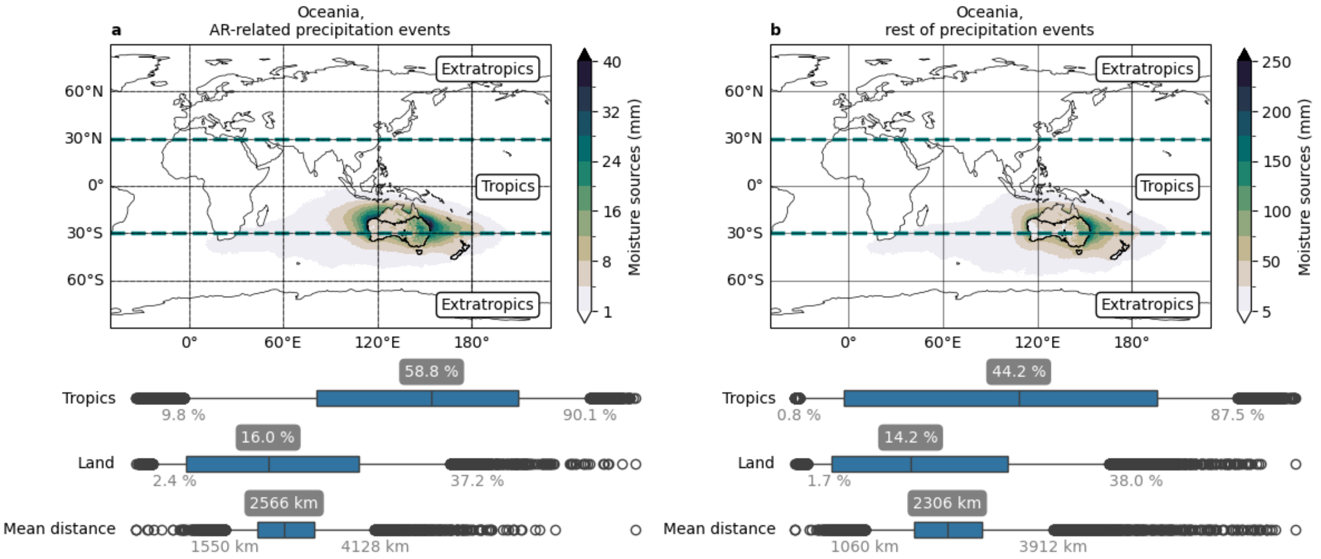

Supplementary Fig. 22 | Precipitation sources with boxplots Oceania. Mean annual accumulated moisture sources for precipitation events affecting Oceania, for AR-related precipitation events (a) and rest of events (b), during the period 1980-2023. Boxplots display the event-level distributions of tropical and continental contributions, as well as the mean source-sink distance; dots indicate values outside the 5th-95th percentile range.

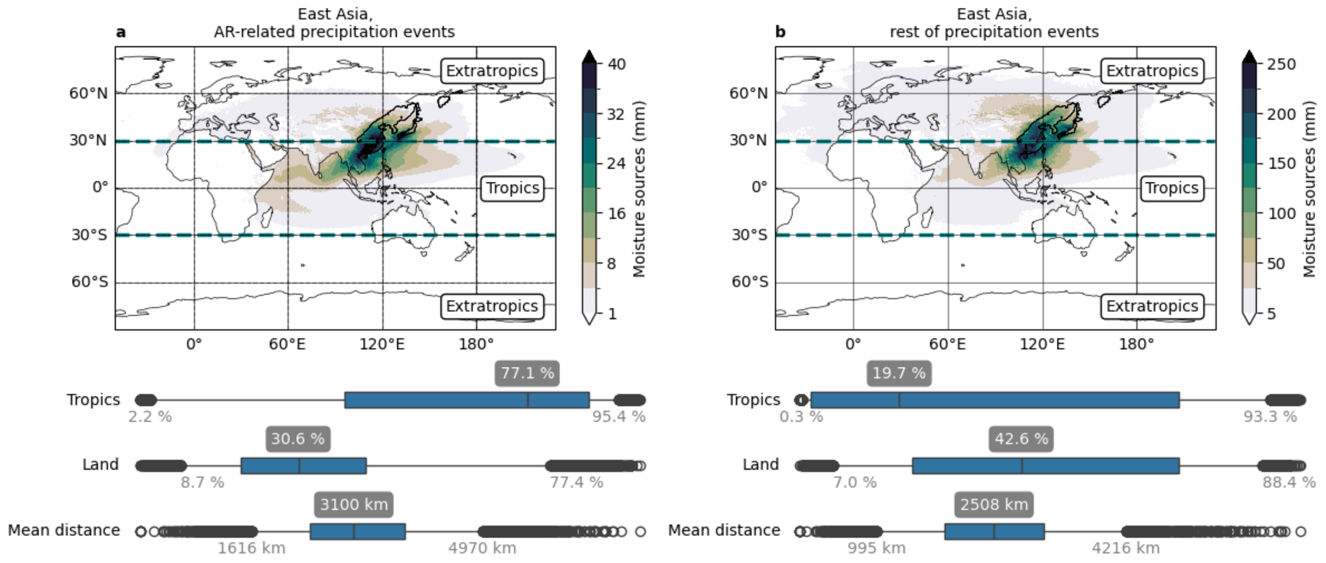

Supplementary Fig. 23 | Precipitation sources with boxplots East Asia. Mean annual accumulated moisture sources for precipitation events affecting East Asia, for AR-related precipitation events (a) and rest of events (b), during the period 1980-2023. Boxplots display the event-level distributions of tropical and continental contributions, as well as the mean source-sink distance; dots indicate values outside the 5th-95th percentile range.

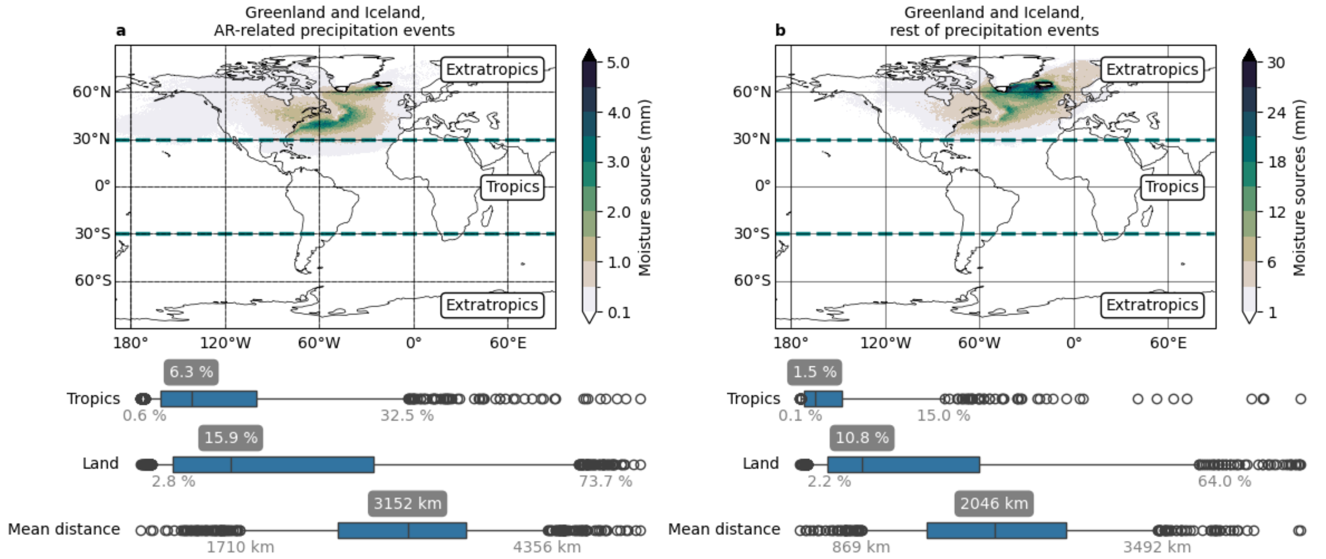

Supplementary Fig. 24 | Precipitation sources with boxplots Greenland and Iceland. Mean annual accumulated moisture sources for precipitation events affecting Greenland and Iceland, for AR-related precipitation events (a) and rest of

events (b), during the period 1980-2023. Boxplots display the event-level distributions of tropical and continental contributions, as well as the mean source-sink distance; dots indicate values outside the 5th-95th percentile range.

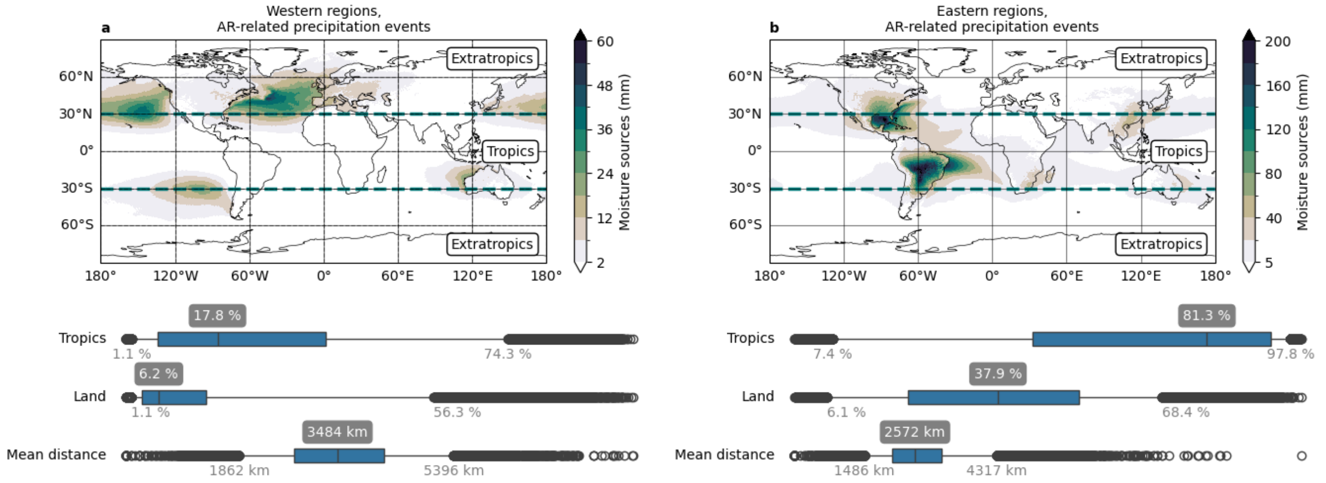

Supplementary Fig. 25 | Precipitation sources with boxplots western and eastern regions. Mean annual accumulated moisture sources for precipitation events affecting western (a) and eastern (b) regions during the period 1980-2023. Boxplots display the event-level distributions of tropical and continental contributions, as well as the mean source-sink distance; dots indicate values outside the 5th-95th percentile range.

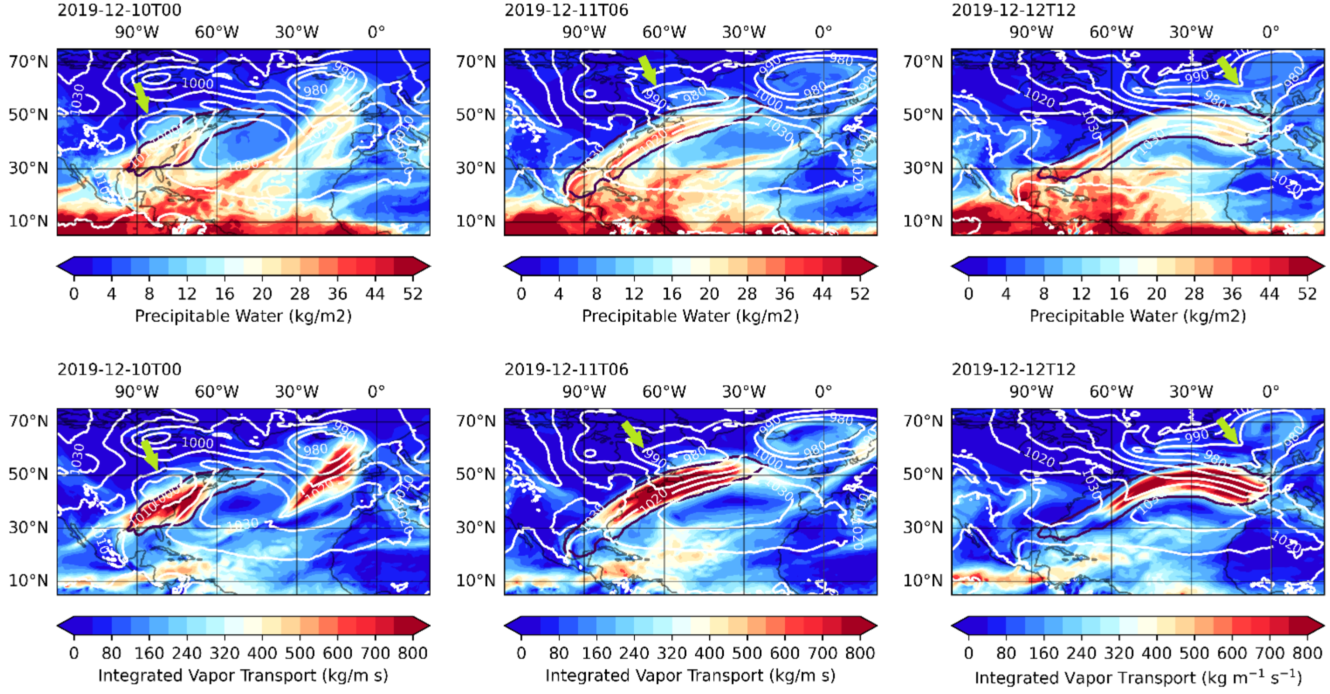

Supplementary Fig. 26 | Real case of AR life cycle. Precipitable water, integrated vapor transport, mean sea level pressure and AR detections (from CG-Climate) for an AR case that first affected eastern North America and then western Europe in December 2019. The role of a high pressure system during the AR's formation stage is evident, as is that of an extratropical cyclone (marked with an arrow) with which it remains associated until reaching Europe.
